# Biochemical characterization of Ty1 retrotransposon protease

**DOI:** 10.1371/journal.pone.0227062

**Published:** 2020-01-09

**Authors:** Lívia Diána Gazda, Krisztina Joóné Matúz, Tibor Nagy, János András Mótyán, József Tőzsér

**Affiliations:** 1 Department of Biochemistry and Molecular Biology, Faculty of Medicine, University of Debrecen, Debrecen, Hungary; 2 Department of Applied Chemistry, Faculty of Science and Technology, University of Debrecen, Debrecen, Hungary; NCI at Frederick, UNITED STATES

## Abstract

Ty1 is one of the many transposons in the budding yeast *Saccharomyces cerevisiae*. The life-cycle of Ty1 shows numerous similarities with that of retroviruses, *e*.*g*. the initially synthesized polyprotein precursor undergoes proteolytic processing by the protease. The retroviral proteases have become important targets of current antiretroviral therapies due to the critical role of the limited proteolysis of Gag-Pol polyprotein in the replication cycle and they therefore belong to the most well-studied enzymes. Comparative analyses of retroviral and retroviral-like proteases can help to explore the key similarities and differences which may help understanding how resistance is developed against protease inhibitors, but the available information about the structural and biochemical characteristics of retroviral-like, and especially retrotransposon, proteases is limited. To investigate the main characteristics of Ty1 retrotransposon protease of *Saccharomyces cerevisiae*, untagged and His_6_-tagged forms of Ty1 protease were expressed in *E*. *coli*. After purification of the recombinant proteins, activity measurements were performed using synthetic oligopeptide and fluorescent recombinant protein substrates, which represented the wild-type and the modified forms of naturally occurring cleavage sites of the protease. We investigated the dependence of enzyme activity on different reaction conditions (pH, temperature, ionic strength, and urea concentration), and determined enzyme kinetic parameters for the studied substrates. Inhibitory potentials of 10 different protease inhibitors were also tested. Ty1 protease was not inhibited by the inhibitors which have been designed against human immunodeficiency virus type 1 protease and are approved as antiretroviral therapeutics. A quaternary structure of homodimeric Ty1 protease was proposed based on homology modeling, and this structure was used to support interpretation of experimental results and to correlate some structural and biochemical characteristics with that of other retroviral proteases.

## Introduction

The transposons of yeast, the Ty elements, are long terminal repeat (LTR)-containing retrotransposons. The LTR-containing class of retrotransposons can be subdivided into the *Ty1-copia* and the *Ty3-gypsy* main classes. The genome of the budding yeast *Saccharomyces cerevisiae* genome contains several retrotransposons, of which the Ty1 retrotransposon is the most well-studied [1, 2].

**Fig 1:**
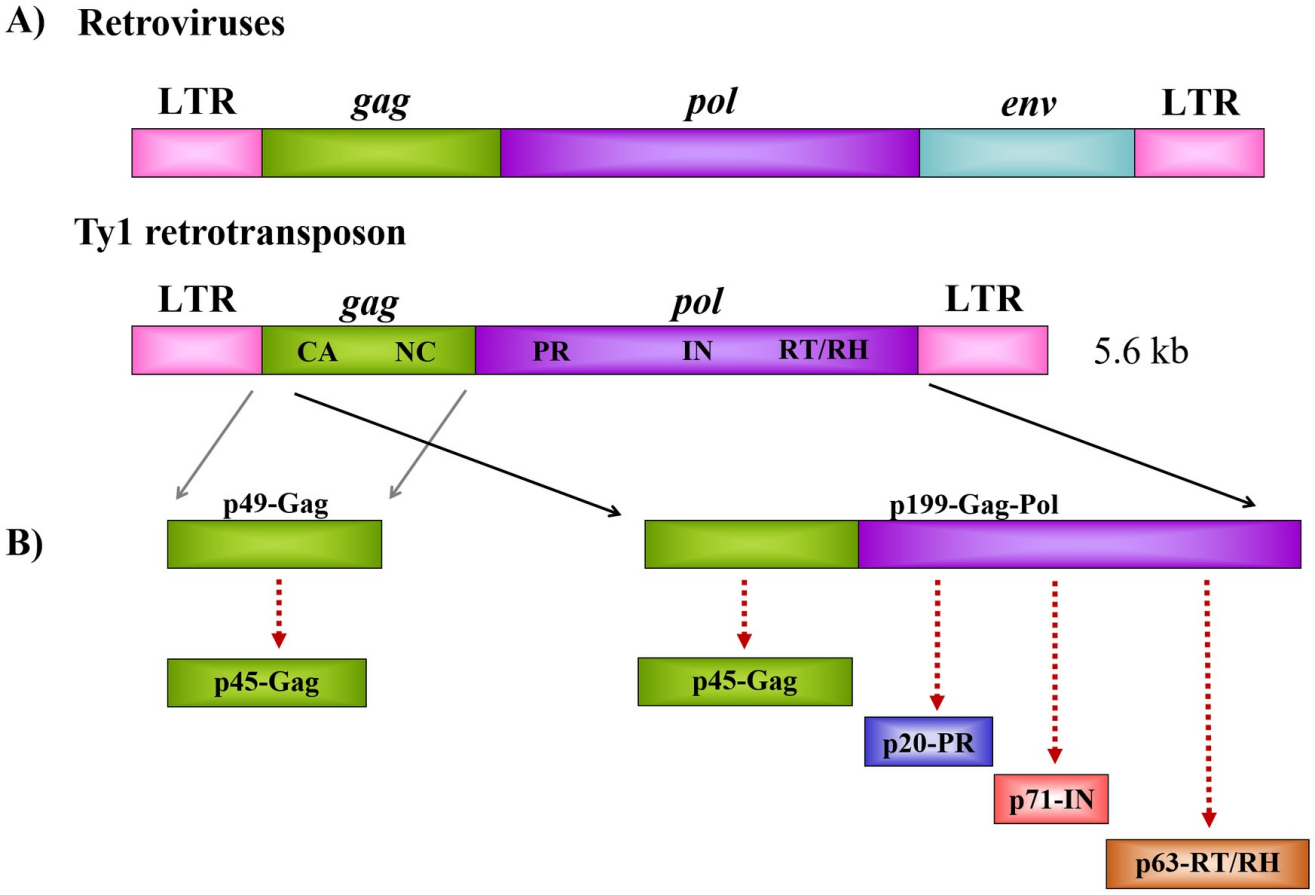
Genome organization of retroviruses and Ty1 retrotransposon. (A) Schematic representations of retroviral and Ty1 retrotransposon genomes are shown. (B) Proteins translated from *gag* and *gag-pol* ORFs are shown. Red dashed lines indicate polyprotein processing by Ty1 PR. Numbers denote molecular weights of the protein products.

Ty1 belongs to the class of LTR-containing retrotransposons which comprise a large family of elements in eukaryotic nuclear genomes, and are highly similar to that of simple retroviruses (Fig 1A). Each end of the Ty1 genome is terminated by identical LTR sequences, and it contains open reading frames (ORF) of *gag* and *pol*, or a single *gag-pol* [1]. Ty1 mRNA contains a 7-nucleotide signal for directing +1 ribosomal frameshifting from the ORF of *gag* to that of *pol* [3, 4]. The proteins which are necessary for retrotransposition are encoded by the genome; while Gag precursor protein (p49-Gag) is translated from *gag*, the Gag-Pol precursor polyprotein (p199-Gag-Pol) is synthesized when frameshifting occurs (Fig 1B). Similarly to retroviruses, limited proteolysis of these precursor polyproteins is a key step of the replication cycle and is carried out by Ty1 PR. The structural proteins and enzymes are processed from Gag or Gag-Pol [5–7], and this cleavage releases p45-Gag, protease, integrase (IN) and reverse transcriptase (RT), products that are similar to the proteins found in retroviruses [8, 9] (Fig 1B).

LTR-containing retrotransposons and retroviruses show similarities in their life-cycle, but due to the lack of obligatory extracellular steps, the replication cycle of the Ty1 retrotransposon is intracellular and is not infectious [2]. This is caused by the lack of *env* gene in the retrotransposon genome (Fig 1A). The Ty1 mRNA, Gag, and Gag-Pol assemble into virus-like particles (VLPs) which undergo intracellular maturation [10, 11]. After maturation of Ty1 proteins, cDNA is synthesized by reverse transcription of Ty1 RNA, followed by nuclear import and integration of the cDNA into the genome by IN enzyme [12–16]. After integration, the life-cycle may start again.

Despite the expanding knowledge about retroviral-like proteases, the information about the biochemical, enzymatic, and structural characteristics of retrotransposon proteases are still limited. The only retrotransposon protease of which the recombinant form has been purified and characterized in detail is the protease of *Drosophila melanogaster*: Copia Gag precursor protein [17]. Both Copia of *D*. *melanogaster* and Ty1 of *S*. *cerevisiae* belong to the Copia transposon endopeptidase family (family A11) based on the MEROPS database [18], but they are distantly related members within this family [19].

The processing pathway and the role of Ty1 PR in VLP formation were already explored [8, 20–22]. It is known that Ty1 PR shares the consensus D-S/T-G-A catalytic motif with retroviral aspartic proteases, and that it processes the functional proteins from the Gag-Pol polyprotein, which is necessary to form functional intracellular VLPs. The proteolysis occurs differentially at the known cleavage sites, which have been identified previously by chemical sequencing [20, 21]. The precursor is cleaved first at the Gag/PR cleavage site, and this processing is highly temperature-sensitive [23]. The structures of any Ty retrotransposon proteases have not been solved experimentally, and preparation of homology model structures was also not published so far, thus structural similarities and differences of Ty retrotransposon and retroviral proteases remained unknown. A recently discovered feature of the human immunodeficiency virus type 1 (HIV-1) PR is the extended surface binding site. The surface residues of the enzyme were found to be able to bind P12-P6 and P6’-P12’substrate residues. This surface site is referred to as the substrate-groove [24], but whether the presence of the binding site is specific for HIV-1 PR and for retroviral proteases or is it shared by all retroviral-like proteases has not been studied. It was also found that the hydrophobicity profiles of Ty1 [20] and Ty3 protease cleavage sites [25] show remarkable differences compared to that of retroviral proteases, but the compositions of substrate binding cavities have not been determined in the case of any Ty retrotransposon protease.

Despite the availability of a protocol for enzyme purification [22], some characteristics of the purified Ty1 PR of *S*. *cerevisiae* has not been investigated so far, therefore, here we aimed to investigate enzymatic properties of Ty1 retrotransposon protease and to compare its properties to that of retroviral and retroviral-like proteases. For characterization, we performed enzyme reactions using different substrates and reaction conditions. Sensitivity of Ty1 PR against protease inhibitors has not been tested previously; therefore, a panel of inhibitors was used to test their inhibitory potential. Besides *in vitro* experiments, *in silico* structural analysis was also performed, homology modeling was used to build a proposed quaternary structure of Ty1 PR. The model was compared to the structures of retroviral and retroviral-like proteases in order to correlate our findings with the results of *in vitro* experiments.

## Materials and methods

### Cloning, protein expression and purification of Ty1 protease

The pET11a vector encoding Ty1 Gag-PR-His_6_ (and containing modified frameshift site in the *gag-pol* overlap region to provide expression of Gag-PR) was a kind gift provided by Dr. J.F. Lawler (The Johns Hopkins University School of Medicine, Baltimore, Maryland, USA). The coding sequence of untagged Ty1 protease (543 bp) was cloned into pET11a plasmid (Novagen) by PCR (see S1 Table for oligonucleotide sequences) from Ty1 Gag-PR-His_6_ using BamHI and NdeI restriction endonucleases. High-Speed Plasmid Mini Kit (Geneaid) was used for plasmid preparation. Both Ty1 PR and Ty1 Gag-PR-His_6_ sequences cloned into pET11a plasmids were sequenced by using BigDye Terminator v3.1 Cycle Sequencing Kit (Applied Biosystems) and data were evaluated using ABI Prism 3100-Avant Genetic Analyzer (Applied Biosystems).

Purified plasmids were transformed into BL21(DE3) *Escherichia coli* cells. Bacteria were grown in 100 ml Luria-Bertani (LB) medium containing 0.1% (w/v) ampicillin at 37°C until reaching an optical density of 0.6–0.8 at 600 nm. Protein expression was induced by the addition of 1.0 mM isopropyl β-D-1-thiogalactopyranoside (IPTG), followed by incubation for 4 h at 26°C. Cells were harvested by centrifugation at 6000 g for 20 min at 4°C using a Thermo Scientific Sorvall Lynx 4000 centrifuge. Following the removal of the supernatant, the pellet was solubilized in 10 ml lysis buffer (20 mM Tris-HCl, 5 mM imidazole, 0.5 mM NaCl, 10% glycerol, pH 8.0) [22] and lysed by sonication for 9 min on ice. Samples were centrifuged at 12000 g for 20 min at 4°C. While Ty1 Gag-PR-His_6_ fusion protein was purified from the soluble supernatant fraction, Ty1 PR was isolated from the insoluble pellet fraction which was suspended in 5 ml of guanidine solution (50 mM Tris-HCl, 6 M guanidine-HCl, pH 8.0).

The untagged Ty1 PR was purified by gel filtration on a Superose 12 10/300 GL column (GE Healthcare) with Äkta Purifier (Amersham Pharmacia Biotech, Uppsala, Sweden) system. The Ty1 Gag-PR-His_6_ fusion protein was purified by Ni-chelate affinity chromatography on a His-Trap Column (GE Healthcare) with Äkta Prime instrument (Amersham Pharmacia Biotech). Purity of the proteases was confirmed by SDS-PAGE using a 14% polyacrylamide gel. The purified fractions were dialyzed against a”yeast *in vivo*-like” buffer (50 mM phosphate buffer, 300 mM KCl, 245 mM glutamate, 50 mM MgSO_4_, 0.5 mM CaCl_2_, 100 mM NaCl, pH 6.8) [26] for 16 h at 4°C, and concentrated by using 10K and 3K Amicon Ultra 0.5 ml centrifugal filters (Merck, Millipore). Protein concentration was determined using the Bradford assay (Sigma).

### Western blotting

Proteins were separated by SDS-PAGE using 14% polyacrylamide gel, and then transferred onto nitrocellulose membrane at 100 V for 70 min. ProSieve PreStained Protein Ladder Plus (Lonza) marker was used as standard. Dry milk (2%) dissolved in Tris-buffered saline (TBS, pH 7.5) was used to block the membrane for 1 h at room temperature. For the detection of tagged Ty1 proteins (Gag-PR-His_6_ and PR-His_6_) by Western blot, we used mouse anti-His primary antibody (460693, Invitrogen) in a 1:5000 dilution (0.24 μg/ml) diluted with TTBS (TBS complemented with Tween20) containing 0.1% dry milk. The membrane was incubated with the primary antibody for 2 h at room temperature. Then, it was washed three times with TTBS for 15 min and followed by incubation with goat anti-mouse secondary antibody (A4416, Sigma) for 1 h at room temperature. After repeated washing steps (in TTBS), the proteins were detected on the membrane by using SuperSignal West Pico chemiluminescent substrate (Thermo Scientific).

### Peptide-based proteolytic assays

The oligopeptides used in the proteolytic assays (Table 1A) were synthesized as previously described [27]. Reaction mixtures contained 10 μl peptide buffer A (20 mM PIPES, 100 mM NaCl, 0.5% Nonidet P-40, 10% glycerol, 2 mM dithiotreitol (DTT), pH 7.0), 5 μl purified Ty1 PR-His_6_ (processed from Ty1 Gag-PR-His_6_) or Ty1 PR, and 5 μl oligopeptide substrate (2 mg/ml) dissolved in water. Reaction mixtures were incubated for the different experiments from 2 up to 5 h at 30°C. The reactions were stopped by the addition of 180 μl 1% trifluoroacetic acid (TFA).

**Table 1:** Kinetic parameters of Ty1 PR.

**A**	**Oligopeptide**	**Enzyme**	**K**_**m**_ (mM)	**k**_**cat**_ (s^-1^)	**k**_**cat**_**/K**_**m**_ (mM^-1^ s^-1^)	
	VPTIN*NVHTS	Ty1 PR	0.21 ± 0.07	0.006 ± 0.001	0.028 ± 0.0105	
	VPTIN*NVHTS	Ty1 PR-His_6_	0.25 ± 0.09	0.009 ± 0.001	0.036 ± 0.0132	
	IHLIA*AVKAV	Ty1 PR, Ty1 PR-His_6_	no cleavage was observed			
	TARAH*NVSTS	Ty1 PR, Ty1 PR-His_6_	no cleavage was observed			
**B**	**Oligopeptide**	Enzyme*	**K**_**m**_ (mM)	**k**_**cat**_ (s^-1^)	**k**_**cat**_**/K**_**m**_ (mM^-1^ s^-1^)	
	VPTIN*NVHTS	HFV PR	0.03 ± 0.01	0.0002 ± 0.00003	0.007 ± 0.0024	
	IHLIA*AVKAV	HFV PR	1.00 ± 0.16	0.0020 ± 0.00020	0.002 ± 0.0004	
	TARAH*NVSTS	HFV PR	not hydrolyzed			
**C**	**Protein substrate**	**Cleavage site sequence**	**K**_**m**_ (μM)	**k**_**cat**_ (s^-1^) (*10^−4^)		**k**_**cat**_**/K**_**m**_ (mM^-1^ s^-1^)
	PR/IN_10aa_wt	VPTIN*NVHTS	4.64 ± 1.52	2.16 ± 0.22		0.047 ± 0.016
	PR/IN_20aa_wt	PSNISVPTIN*NVHTSESTRK	1.90 ± 0.52	2.45 ± 0.19		0.129 ± 0.037
	PR/IN_20aa_mut	GGGGGVPTIN*NVHTSGGGGG	1.90 ± 0.61	2.52 ± 0.17		0.133 ± 0.044
	IN/RT_10aa_wt	IHLIA*AVKAV	1.60 ± 0.78	0.58 ± 0.05		0.036 ± 0.018
	IN/RT_20aa_wt	RSKKRIHLIA*AVKAVKSIKP	3.31 ± 0.65	0.75 ± 0.04		0.023 ± 0.005
	Gag/PR_10aa_wt	TARAH*NVSTS	4.55 ± 1.43	3.20 ± 0.17		0.070 ± 0.022
	Gag/PR_20aa_wt	NSKSKTARAH*NVSTSNNSPS	2.27 ± 0.90	0.66 ± 0.05		0.029 ± 0.012
**D**	**Protein substrate**	**Cleavage site sequence**	**K**_**m**_ (μM)	**k**_**cat**_ (s^-1^) (*10^−4^)		**k**_**cat**_**/K**_**m**_ (mM^-1^ s^-1^)
	PR/IN_10aa_wt	VPTIN*NVHTS	4.50 ± 1.76	19.15 ± 2.5		0.426 ± 0.176
	PR/IN_20aa_wt	PSNISVPTIN*NVHTSESTRK	10.00 ± 1.59	55.80 ± 4.0		0.558 ± 0.097
	PR/IN_20aa_mut	GGGGGVPTIN*NVHTSGGGGG	6.12 ± 1.33	38.00 ± 2.8		0.621 ± 0.142
	IN/RT_10aa_wt	IHLIA*AVKAV	6.96 ± 2.21	20.00 ± 2.0		0.287 ± 0.096
	IN/RT_20aa_wt	RSKKRIHLIA*AVKAVKSIKP	10.81 ± 3.79	30.30 ± 4.7		0.280 ± 0.108
	Gag/PR_10aa_wt	TARAH*NVSTS	18.21 ± 4.58	46.60 ± 6.1		0.256 ± 0.072
	Gag/PR_20aa_wt	NSKSKTARAH*NVSTSNNSPS	7.92 ± 9.11	34.96 ± 11.9		0.441 ± 0.530

(A) Oligopeptide substrates representing natural PR/IN (VPTIN*NVHTS), IN/RT (IHLIA*AVKAV) and Gag/PR (TARAH*NVSTS) cleavage sites of Ty1 retrotransposon were tested as substrates of Ty1 PR and Ty1 PR-His_6_. (B) Values determined previously for HFV PR [40] are also shown for comparison. (C-D) His_6_-MBP-mTurquoise2 recombinant fluorescent protein substrates representing natural PR/IN, IN/RT, or Gag/PR cleavage site of Ty1 retrotransposon were used as substrates for untagged Ty1 PR, the cleavage reactions were performed in cleavage buffer A (C) or cleavage buffer B (D). Errors represent SD (n≥2).

The products were separated by an HPLC-based method using a 0–100% water-acetonitrile gradient in the presence of TFA on Merck Hitachi instrument. For enzyme kinetic measurements, VPTIN*NVHTS oligopeptide substrate (representing Ty1 PR/IN cleavage site) was used at 0.2–1.2 mM; Ty1 PR (400–1600 nM) was incubated with the substrate at 30 °C for 2 h, whereas Ty1 PR-His_6_ (500–1500 nM) was incubated for 2.5 h. Kinetic parameters (shown in Table 1A) were determined by fitting the data obtained at less than 20% substrate hydrolysis to the Michaelis-Menten equation using GraphPad Prism version 5.00 for Windows (GraphPad Software, La Jolla California USA, www.graphpad.com). Statistical significances were determined by GraphPad QuickCalcs (https://www.graphpad.com/quickcalcs/ttest2).

### Dependence of enzyme activity on ionic strength, pH, temperature and urea concentration

To determine the effects of different reaction conditions on protease activity, VPTIN*NVHTS synthetic oligopeptide substrate (0.47 mM) was used as substrate in cleavage reactions. Reactions were performed in peptide buffer B (100 mM MES, 200 mM Tris, 100 mM sodium acetate) for 4 h, assays were initiated by mixing 10 μl buffer, 5 μl substrate, and 5 μl enzyme. The pH optimum of Ty1 PR-His_6_ was determined in peptide buffer B for 4 h at 30°C, the pH range was set to be 6.5–9.0. The effect of ionic strength was also determined in peptide buffer B (pH 8.0), the final concentration of NaCl ranged from 0.5 up to 2 M, reaction mixtures were incubated for 5 h at 30°C. The temperature optimum of the Ty1 protease was determined in peptide buffer A, the temperature ranged from 18 to 37°C. Urea dissociation curve was determined in peptide buffer B (pH 8.0), buffer was supplemented with urea (from 0.05 up to 0.25 M), and the incubation was performed for 4 h at 30°C.

### Inhibition study

Ty1 PR-His_6_ activity was measured in the presence of various HIV-1 protease inhibitors. Atazanavir, nelfinavir, saquinavir, darunavir, amprenavir, lopinavir, tipranavir [28], acetyl-pepstatin, and pepstatin A [29], and DMP-323 [30] were in-house stocks. To study effect of inhibitors on proteolytic activity, reaction mixtures contained 5 μl purified Ty1 PR-His_6_ (400–1600 nM), 10 μl peptide buffer A, 4.8 μl oligopeptide substrate (VPTIN*NVHTS, 0.44 mM), and 0.2 μl inhibitor. For screening, inhibitors were dissolved in DMSO to have ≥100 nM final concentration. DMSO solution containing no inhibitor was used as control. Reaction mixtures were incubated for 2 h at 30°C. The half maximal inhibitory concentration (IC_50_) was determined for acetyl-pepstatin (final concentration ranging from 100 up to 1000 nM).

### Expression vector for fluorescent kinetic assays

We used a slightly modified pDest-His_6_-MBP-mTurquoise2 plasmid, prepared in our laboratory by Gateway Cloning Technology as previously described [31], and modified in the present study as follows. The empty pDest-His_6_-MBP-mTurquoise2 plasmid was linearized by PacI and NheI endonucleases (New England Biolabs). After separation by electrophoresis, the linear plasmid was extracted from a 1% agarose gel by NucleoSpin Gel and PCR Clean-up kit (Macherey-Nagel). The oligonucleotides containing a BamHI restriction site prior to the coding sequence of a (GGGGS)_4_ flexible linker (S1 Table) were incubated with the purified linear pDest-His_6_-MBP-mTurquoise2 plasmid (150 ng). For annealing, the mixture was incubated for 2 min at 65°C then 2 min at 4°C. After the addition of T4 DNA ligase and T4 DNA ligase buffer (10X) (New England Biolabs), the mixture was incubated for 16 h at 16°C. Then, the reaction mixture (5 μl) was transformed by heat shock into TOP10 *E*. *coli*-derived competent cells, followed by spread and growth on selective LB agar plates (containing ampicillin). After culturing of the selected colonies, plasmids were purified using High-Speed Plasmid Mini Kit (Geneaid) and later sequenced by using BigDye^®^ Terminator v3.1 Cycle Sequencing Kit (Applied Biosystems) and capillary DNA sequencing using a sequencing forward primer (S1 Table).

For the cloning of the cleavage site’s coding sequences into the expression vector, a pDest-His_6_-MBP-(GGGGS)_4_-mTurquoise2 plasmid was linearized with BamHI and PacI restriction endonucleases (New England Biolabs). The linear plasmid was separated by electrophoresis and purified from the 1% agarose gel using NucleoSpin Gel and PCR Clean-up kit (Macherey-Nagel). Annealing and ligation were performed as described above in this section, using 150 ng purified linear plasmid and 200 ng oligonucleotide primer (S1 Table) for each reaction.

### Expression and purification of fluorescent substrates

Recombinant fluorescent substrates were expressed in *E*. *coli* BL21(DE3) cells as previously reported [31–33]. The His_6_-tagged fluorescent recombinant protein substrates were purified from the supernatant of the lysed cells by the addition of Ni-NTA magnetic agarose beads (Qiagen) and were incubated for 30 min while continuously shaking. Using a Dynamag^™^-2 magnetic particle concentrator (Thermo Fischer Scientific, Invitrogen), the magnetic beads were washed with washing buffer (50 mM sodium-acetate, 300 mM NaCl, 5 mM imidazole, 0.05% Tween20, pH 7.0). Finally, the magnetic beads binding the His_6_-tagged fluorescent recombinant protein substrates were washed with the following Ty1 cleavage buffers: cleavage buffer A (10 mM PIPES, 75 mM NaCl, 0.25% Nonidet P-40, 5% glycerol, 75 mM KCl, 12.5 mM NaH_2_PO_4_, 61.25 mM sodium-glutamate, 12.5 mM MgSO_4_, 0.125 mM CaCl_2_, 0.05% Tween20, pH 7.0) or cleavage buffer B (50 mM MES, 100 mM Tris, 50 mM sodium-acetate, 150 mM NaCl, 75 mM KCl, 12.5 mM NaH_2_PO_4_, 61.25 mM sodium-glutamate, 12.5 mM MgSO_4_, 0.125 mM CaCl_2_, 0.05% Tween20, pH 8.0). The purified His_6_-tagged fluorescent recombinant protein substrates were used for proteolytic assays, based on the method described previously [31–33].

### In-solution digestion and gel electrophoretic analysis

The recombinant substrates were purified for in-solution digestion by their elution from the affinity beads using elution buffer (100 mM EDTA, 0.05% Tween20, pH 8.0), followed by buffer exchange to distilled water using 10K Amicon Ultra-0.5 mL centrifugal filters (Millipore). The reaction mixtures contained 10 μl peptide buffer A, 5 μl recombinant protein substrate (1–3 mg/mL), and 5 μl Ty1 PR (300–1200 nM), while control samples contained”yeast *in vivo*-like” buffer in place of enzyme. The cleavage reactions were incubated for 16 h at 30 °C and stopped by the addition of Laemmli sample buffer (containing SDS and β-mercaptoethanol). Before electrophoresis, proteins were denatured at 95°C for 7 min. Uncleaved substrates and cleavage products were separated by SDS-PAGE using 16% SDS gels. The denatured fluorescent proteins were renatured by rinsing the polyacrylamide gel in distilled water to remove SDS, as described previously [31, 33]. After in-gel renaturation, both blue light transillumination (Dark Reader transilluminator, Clare Chemical Research) and Coomassie staining (PageBlue Protein Staining solution, Thermo Scientific) were used for protein detection.

### Fluorescent assay of proteolysis and calibration curve of recombinant fluorescent substrates

To assay the kinetics of proteolysis, Ni-NTA beads were coated with substrates as follows. A homogenous suspension of His_6_-tagged substrates was assayed in 2.0 ml Protein Lobind Microcentrifuge tubes (Eppendorf) using increasing concentrations of the substrate. The supernatant was removed using a Dynamag^™^-2 magnetic particle concentrator, and cleavage buffer A or B was added to set equal final volume for each sample. After the reaction, substrate concentrations were determined using the Bradford assay. Substrate control samples and blanks were also prepared in the same manner to allow for determination of concentrations and to detect non-specific substrate dissociation. To determine the substrate concentration, blank samples were incubated in elution buffer in parallel with the kinetic measurements. Kinetic measurements were carried out by the cleavage of His_6_-MBP-VPTIN*NVHTS-(GGGS)_4_-mTurquoise2 (PR/IN_10aa_wt), His_6_-MBP-PSNISVPTIN*NVHTSESTRK-(GGGS)_4_-mTurquoise2 (PR/IN_20aa_wt), His_6_-MBP-GGGGGVPTIN*NVHTSGGGGG-(GGGS)_4_-mTurquoise2 (PR/IN_20aa_mut), His_6_-MBP-TARAH*NVSTS-(GGGS)_4_-mTurquoise2 (Gag/PR_10aa_wt), His_6_-MBP-NSKSKTARAH*NVSTSNNSPS-(GGGS)_4_-mTurquoise2 (Gag/PR_20aa_wt), His_6_-MBP-IHLIA*AVKAV-(GGGS)_4_-mTurquoise2 (IN/RT_10aa_wt), and His_6_-MBP-RSKKRIHLIA*AVKAVKSIKP-(GGGS)_4_-mTurquoise2 (IN/RT_20aa_wt) substrates (up to 0.08 mM concentration) by untagged Ty1 PR (up to micromolar concentrations). Reaction mixtures were incubated at 30°C for 2 h. The enzyme reactions were stopped by separation of the supernatants from the magnetic beads. Fluorescence of supernatants was measured using a Synergy2 multimode plate reader, using 400/10 nm excitation and 460/40 nm emission filters. The relative fluorescent intensity (RFU) values were corrected by that of the blank samples, then divided by the slopes of the substrate calibration curves in cleavage buffers, and were plotted against the concentration of coated substrates (μM). The substrate control samples were used to determine the substrate concentration by dividing the RFU by the slope of the substrate calibration curve in the elution buffer. Kinetic parameters were determined at less than 20% substrate hydrolysis by Michaelis-Menten non-linear regression analysis using GraphPad Prism version 5.00 for Windows (GraphPad Software, La Jolla, California USA, www.graphpad.com) (Table 1).

### *In silico* analyses

Secondary structure prediction was performed by using the PredictProtein server [34] based on the sequence of Ty1 PR (UniProtKB: Q07793). Disorder prediction was performed using the IUPred web server [35]. Crystal structures of yeast DNA damage-inducible protein 1 (Ddi1) (PDBID: 2I1A) [36] and xenotropic murine leukemia virus-related virus (XMRV) protease (PDBID: 4EXH) [29] were used as templates for homology modeling by Modeller 9v13 [37]. Molecular visualizations were performed using the PyMOL Molecular Graphics System (Version 1.3 Schrödinger, LLC).

### Sample preparation for cleavage site identification

The Ni-NTA magnetic beads were coated with the recombinant substrates and then incubated with Ty1 PR in cleavage buffer B at 30°C for 16 h. After the incubation, cleavage products were eluted from the beads by imidazole-containing buffer (50 mM NaH_2_PO_4_, 300 mM NaCl, 250 mM imidazole, pH 8.0). The eluted fractions were concentrated by repeated centrifugation steps (12000 × g, 10 cycles) using 10K Amicon Ultra 0.5 ml centrifugal filters while changing the buffer to 50 mM Tris (pH 8.0). TEV PR was added to the concentrated samples, followed by incubation at 30°C for 16 hours. TEV PR stock solution was a kind gift of David S. Waugh (NCI-Frederick, USA), and was purified by the method described previously [38]. After incubation, the samples were analyzed by MALDI-TOF MS in order to determine the molecular weights of the short proteolytic fragments released upon cleavage by Ty1 and TEV PRs.

### Matrix-assisted laser desorption/ionization time-of-flight mass spectrometry (MALDI-TOF MS)

The MALDI-TOF MS measurements were carried out by a Bruker Autoflex Speed mass spectrometer. Reflectron mode was used for all samples, where the reflectron voltage 1, reflectron voltage 2, ion source voltage 1 and ion source voltage 2 were 21.00 kV, 9.55 kV, 19.00 kV and 16.65 kV, respectively. Solid phase laser (355 nm, ≥100 μJ/pulse) was applied at 500 Hz and 10 000 shots were summed. Spectra were calibrated by Peptide Calibration Standard obtained from Bruker.

The samples were prepared with 2,5-dihydroxybenzoic acid (DHB) matrix dissolved in 50% aqueous acetonitrile with 0.1% TFA, the concentration was 100 mg/ml. 1 μl matrix was deposited on the plate and 1 μl sample was added immediately and allowed to dry.

## Results

### Cloning, expression, and purification of Ty1 protease

**Fig 2:**
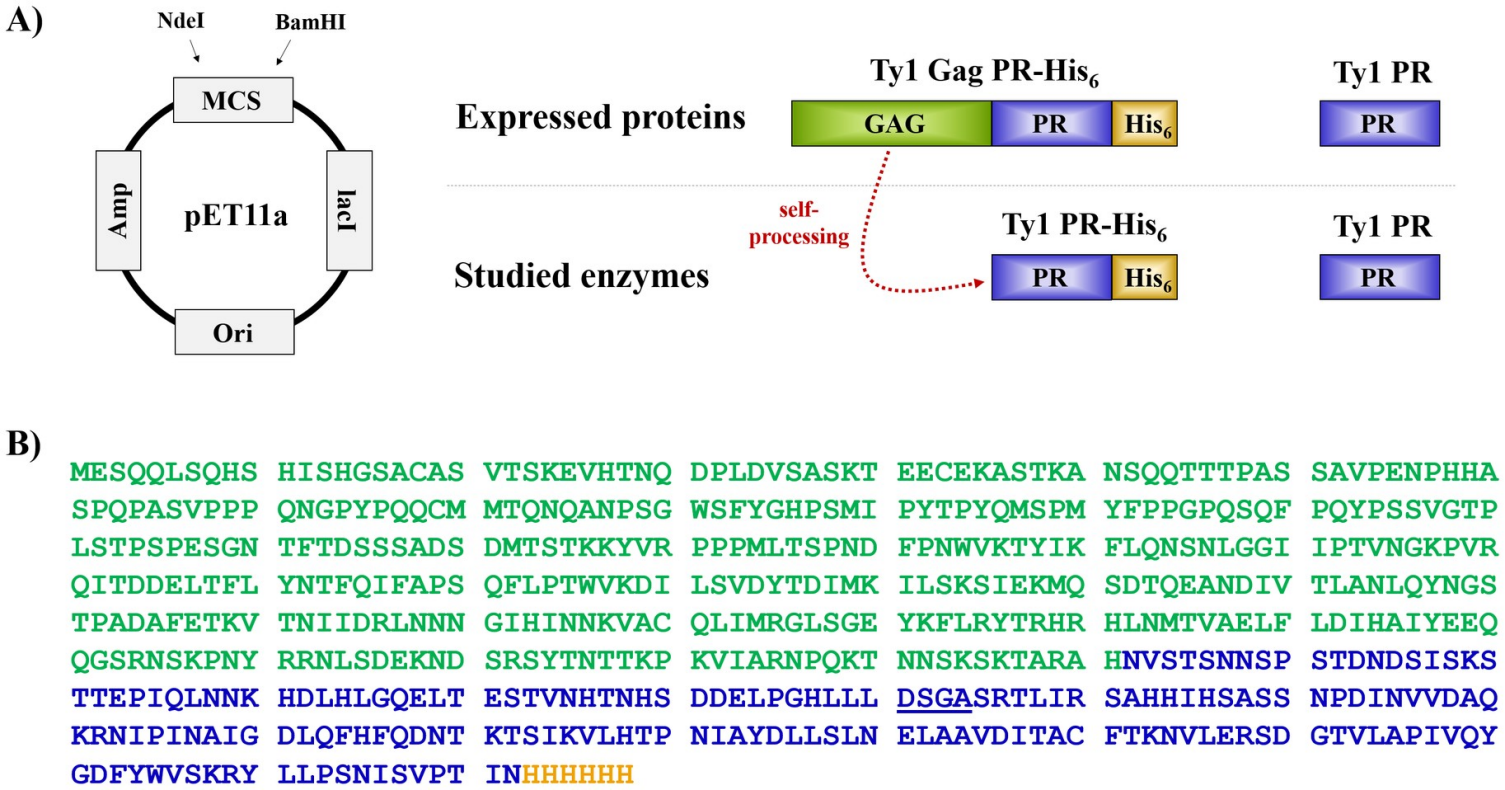
Studied forms of Ty1 protease. (A) Coding sequences were cloned into pET11a bacterial expression plasmid using NdeI and BamHI restriction endonucleases. The Ty1 Gag-PR-His_6_ precursor polyprotein and the untagged protease have been expressed in bacterial cells. The Ty1 PR-His_6_ is formed by the autoproteolysis (dashed red line) of Gag-PR-His_6_ precursor protein. Abbreviations: Amp, ampicillin resistance gene; lacI, repressor protein gene; Ori, bacterial origin; MCS, multi cloning site. (B) Sequence of Ty1 Gag-PR-His_6_ is shown. Color code: Gag, green; protease, blue; His_6_, orange. Active site motif is underlined.

**Fig 3:**
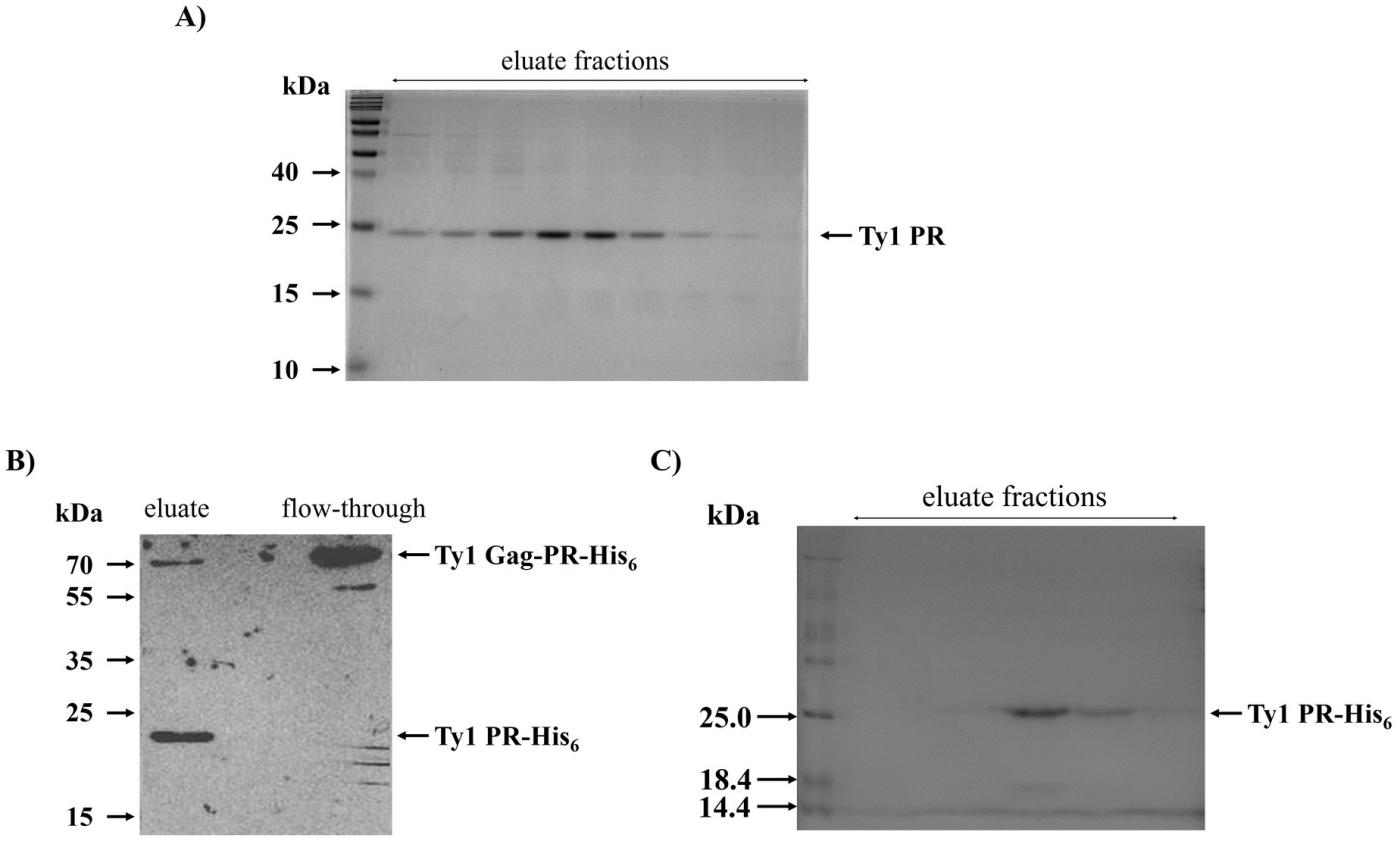
Purification of Ty1 PR and Ty1 PR-His_6_. (A) Image of a Coomassie-stained SDS-PAGE gel of eluate fractions after purification of untagged Ty1 PR by gel filtration. (B) Western blot image shows that Ty1 PR-His_6_ (molecular weight: ~21 kDa) was processed from its Gag-PR-His_6_ precursor (molecular weight: ~72 kDa) during the purification. Both protein forms were detected by anti-His antibody. While the Ty1 PR-His_6_ processed form was detected in the elute fraction, the full-length precursor was present in the flow-through. (C) Image of a Coomassie-stained SDS-PAGE gel showing eluate fractions from Ty1 PR-His_6_ purification. Purified fractions of Ty1 PR-His_6_—which contained no unprocessed Gag-PR-His_6_ precursor—were used for protease assays.

A pET11a plasmid constructed for the bacterial expression of Ty1 Gag-PR-His_6_ was kindly provided by Dr. J.F. Lawler. The coding sequence of Ty1 PR was cloned into a pET11a bacterial expression plasmid, and the success of cloning was verified by sequencing. Plasmids bearing the coding sequences of Ty1 PR or Ty1 Gag-PR-His_6_ (Fig 2) were transformed into *E*. *coli* cells. After refolding, Ty1 PR protease was purified by gel filtration (Fig 3A). The Ty1 Gag-PR-His_6_ recombinant protein was found to be processed, as determined by its purification using Ni-chelate affinity chromatography. Therefore, the autoproteolysis of Ty1 Gag-PR-His_6_ precursor (molecular weight: ~72 kDa) resulted in the Ty1 PR fused to a C-terminal hexahistidine tag (Ty1 PR-His_6_) (molecular weight: ~21 kDa). This result suggests that autoproteolysis occurred, as previously observed for the processing of Gag protein by Ty1 PR [20]. Both the precursor and the processed proteins were identified by Western Blot, thereby proving the presence of the different enzyme forms (Fig 3B). The processed Ty1 PR-His_6_ fusion protein was used for the proteolytic assays (Fig 3C).

### Enzymatic assays using synthetic oligopeptide substrates

For investigation of optimal conditions for enzyme folding, Ty1 PR was dialyzed against various buffers and buffer combinations that have been shown previously to be suitable for retroviral protease activity [39]. In peptide-based assays, both protease and oligopeptide solutions were added to the buffer, and the reaction mixtures were incubated at 30°C, and stopped by the addition TFA. The mixtures were then injected onto a reversed-phase chromatography column in order to separate substrates and cleavage products, the substrate turnover was determined by integration of peak areas.

**Fig 4:**
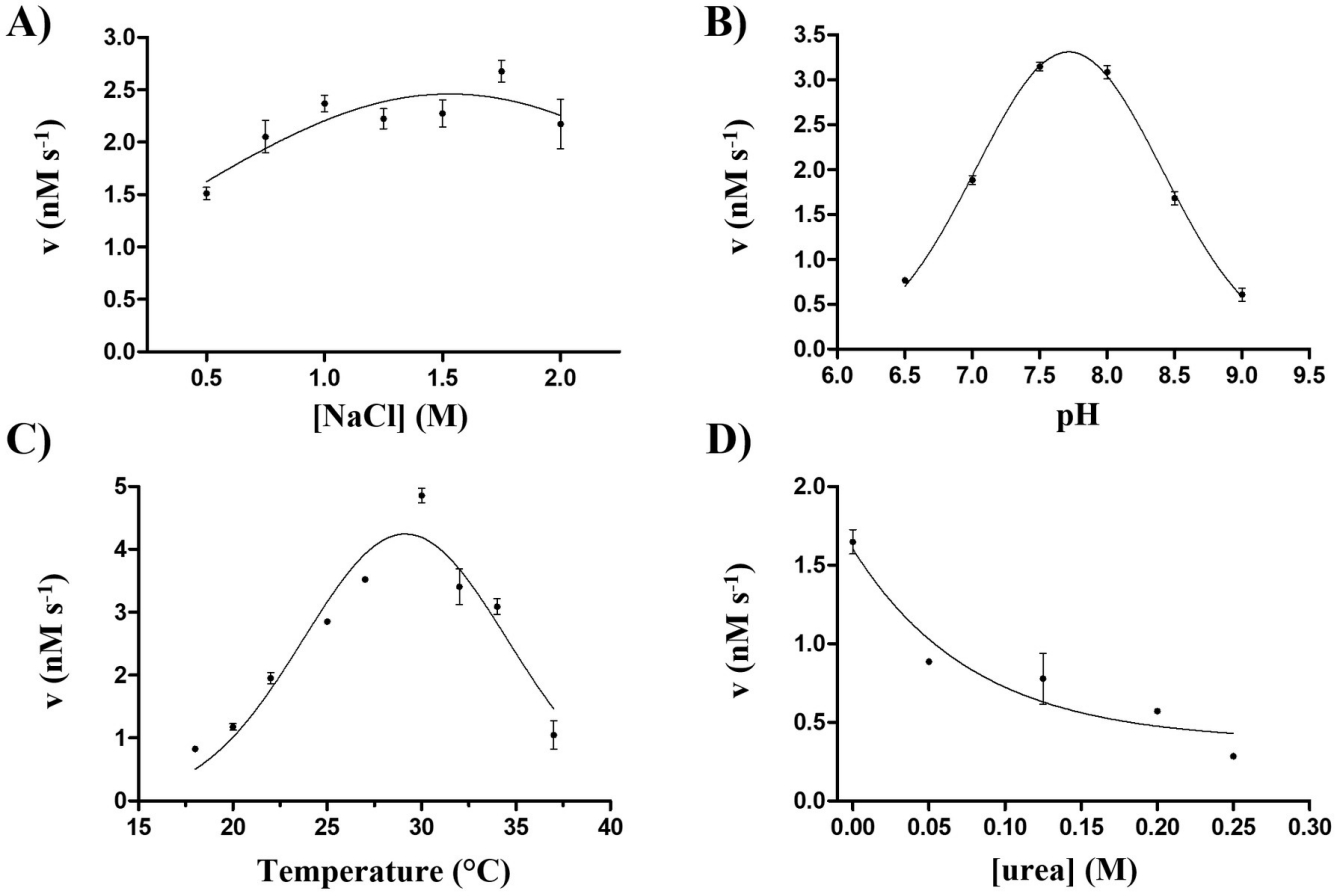
Effects of reaction conditions on Ty1 protease activity. In all measurements the enzyme was incubated with VPTIN*NVHTS synthetic substrate, and reaction velocity was determined (nM s^-1^). (A) Effects of ionic strength on the activity of Ty1 PR-His_6_ measured at increasing NaCl concentrations. (B) Determination of the pH optimum of Ty1 PR-His_6_ in 6.5–9.0 pH range. (C) Determination of the temperature optimum of Ty1 PR. D) Dependence of enzyme activity of Ty1 PR-His_6_ on urea concentration (in 0–0.25 M concentration range). Error bars represent SD (n = 2).

Ty1 PR—dialyzed against”yeast *in vivo*-like” buffer [26]—showed activity only in PIPES- (peptide buffer A) or MES-based (peptide buffer B) buffers. The proportion of different buffers in the reaction mixtures was also found to be a determinant of enzyme activity, and optimal ratio of water,”yeast *in vivo*-like”, and PIPES or MES-based buffers was found to be 1:1:2, respectively. Besides determination of optimal buffer environment, the effects of different reaction conditions have also been investigated to determine the biochemical characteristics of the protease (Fig 4).

The dependence of enzyme activity on ionic strength was studied, and highest activities were measured at 1.5–2 M NaCl concentration (Fig 4A). Similarly to the proteases of HIV-1 and human foamy virus (HFV) [40, 41], the enzyme activity was boosted by high ionic strength, and higher activity was observed for Ty1 PR at > 1 M NaCl concentration. Copia transposon protease of *D*. *melanogaster* also showed highest activity at high (2 M) NaCl concentration; activity was significantly lower at < 2 M NaCl concentration, but higher ionic strengths also decreased activity [17].

pH optimum of Ty1 PR was found to be slightly alkaline (7.7) (Fig 4B), which is higher than that of any studied retroviral proteases. For instance, the optimal pH of HFV protease is 6.6–6.8 [40] while that of HIV-1 PR is between 4 and 6 [41]. Interestingly, the optimal pH required for *D*. *melanogaster* Copia transposon protease was found to be similar to that of HIV-1, with synthetic substrate cleaved most efficiently at pH 4.0 [17].

Temperature optimum was found to be close to 30°C, lower enzyme activities were measured at higher temperatures (Fig 4C). This is comparable with the optimal temperature required for the proteases of some non-retroviral proteases like tobacco vein mottling virus (TVMV) and tobacco etch virus (TEV), which also showed decreased activities at higher temperatures (> 34°C) [42]. In contrast, HFV [40] and HIV-1 PRs [41] were highly active at 37°C. The observed temperature optimum of Ty1 PR is in agreement with the previous findings of Lawler and coworkers who observed significantly lower transposition ability at high temperatures (32–36°C), due to the temperature sensitivity of Ty1 PR [23]. Interestingly, Copia transposon protease of *D*. *melanogaster* was found to have lower temperature sensitivity, with highest activity measured at 70 °C, but relative activity not lower than 50% in the 20–70°C temperature range [17].

The Ty1 protease was observed to be sensitive to urea, increasing the urea concentration caused decrease of enzyme activity (Fig 4D). The urea concentration causing 50% loss of enzyme activity (also referred as urea dissociation constant, UC_50_) for Ty1 PR was found to be 0.05 M. This concentration is markedly lower than that of HIV-1 PR (UC_50_ = 1.47 M) and is more similar to that of XMRV PR (UC_50_ = 0.2 M) [29]. Higher sensitivity to urea implies lower dimer stability for Ty1 protease, the possible structural background of this difference is discussed later in the *In silico structural analysis* section.

Both Ty1 PR and Ty1 PR-His_6_ enzyme forms showed very low specific activities on synthetic oligopeptide substrates representing Ty1 cleavage sites, as compared to the findings with retroviral proteases—especially HIV-1, human T-lymphotropic virus type 1, bovine leukemia virus, and Moloney murine leukemia virus proteases—on peptides representing their respective cleavage sites [43]. Similarly low catalytic activities were also observed previously for HFV and the Gag-encoded Avian myeloblastosis virus (AMV) proteases [40]. Both the untagged, and the His_6_-tagged (self-processed) PRs cleaved the VPTIN*NVHTS synthetic oligopeptide substrate representing the PR/IN cleavage site of Ty1, and the kinetic constants have been determined (Table 1A). The specificity constants were similar, the difference between the obtained values was found to be not significant statistically, which implied a negligible influence of the C-terminal histidine tag, and similar folding efficiency. The very low specificity constants were comparable to that reported for HFV proteinase (0.007 mM^-1^s^-1^) obtained by using this substrate and a buffer optimized for that protease (Table 1B), and cleavage efficiency on peptides representing HFV cleavage sites were found to be very similar to this value [40]. Moreover, we have also tested the cleavage of synthetic oligopeptides representing other Ty1 cleavage sites (IN/RT: IHLIA*AVKAV; Gag/PR: TARAH*NVSTS); however, no cleavage was observed in these substrates (Table 1A).

### Enzymatic assays using recombinant protein substrates

**Fig 5:**
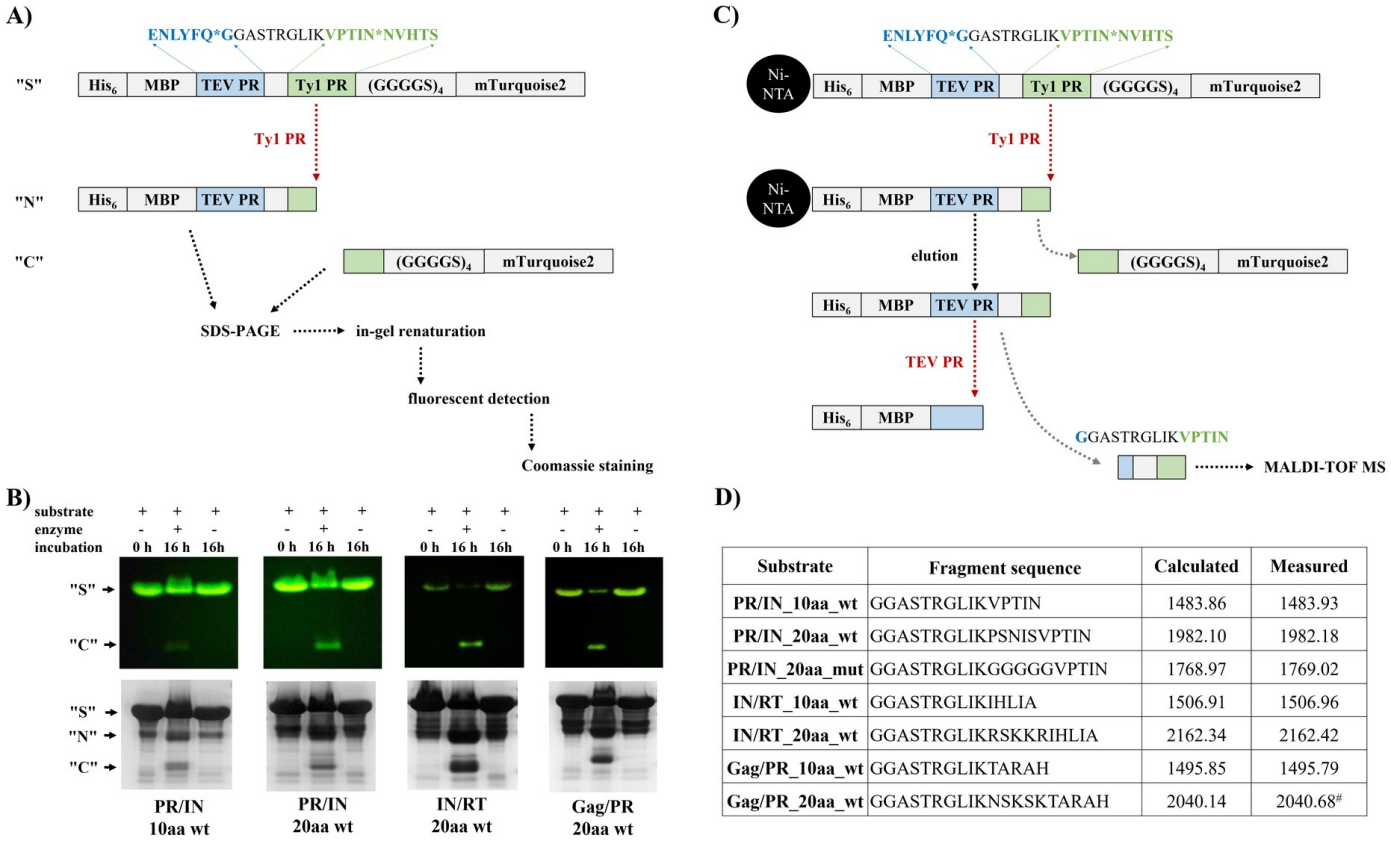
Cleavage reactions by fluorescent recombinant protein substrates. (A) Schematic representation of a recombinant fusion protein substrate and scheme of in-solution cleavage reactions. The TEV PR and Ty1 PR cleavage sites are colored by red and green, respectively. Cleavage site sequences are also shown for both proteases, asterisks indicate cleavage position. Red arrow shows cleavage by Ty1 PR, upon cleavage of the substrate (“S”), N- and C-terminal cleavage products (“N” and “C”, respectively) are produced. After enzymatic digestion, the cleavage products and uncleaved substrates can be separated by denaturing SDS-PAGE. Proteins can be visualized after in-gel renaturation of fluorescent proteins by blue light transillumination or by Coomassie staining. (B) Representative gel images are shown for substrates containing PR/IN, IN/RT, and Gag/PR cleavage sites, after cleavage reactions the bands were visualized in the polyacrylamide gels by blue light transillumination (upper gel images) and by Coomassie staining (lower gel images), as well. (C) The workflow of cleavage site identification, which includes a cleavage reaction with Ty1 PR, the separation of the cleavage fragments, and the digestion of N-terminal cleavage fragment by TEV PR. Resulted short fragments can be subjected to MALDI TOF-MS analysis. The recombinant substrate is identical with that one shown in figure part A, but here we show its immobilization to magnetic affinity beads (Ni-NTA). (D) The molecular weights (Da) of proteolytic fragments were calculated by ProtParam tools of ExPASy (available at https://web.expasy.org/protparam), and were compared to [M+H]^+^ values (Da) determined by MALDI-TOF MS. ^#^ denotes detection with low intensity.

For enzymatic assays, we have applied a previously published recombinant fusion protein substrate-based method [31, 33]. The schematic representation of a recombinant substrate is shown in Fig 5. The protein substrates contain an N-terminal His_6_ affinity tag which enables protein immobilization, the maltose binding protein (MBP) partner improves folding, while the fluorescent tag (mTurquoise2) provides fluorimetric detection. The substrates contain a control cleavage site (for TEV PR), which was used in the identification of cleavage position in the substrate. The substrates contain a cleavage site of the studied protease, as well, the herein designed substrates contained different cleavage site sequences of Ty1 PR (Table 1C and 1D). In this study, we have modified the primarily designed substrate system [31, 33] by the insertion of a (GGGGS)_4_ sequence prior to the fluorescent protein tag. The incorporated sequence is a known flexible linker [44]; besides improving folding, the linker was expected to make the cleavages site more accessible for the protease due to providing higher flexibility for the fluorescent tag.

Seven different recombinant protein substrates, representing naturally occurring cleavage sites of Ty1 PR, were tested in different buffer systems (Table 1C and 1D). Many components were identical in the applied buffers, but the PIPES-based cleavage buffer A had lower pH (7.0) and contained glycerol and Nonidet P-40. The MES-based cleavage buffer B had higher pH (8.0) and ionic strength, conditions found to be optimal for activity of Ty1 PR on the peptide substrate (Fig 4). Catalytic constants were considerably higher when determined in cleavage buffer A (Table 1C) than in cleavage buffer B (Table 1D).

To detect the uncleaved substrates and cleavage fragments in the reaction mixtures, the samples were analysed by SDS-PAGE. We observed no significant unspecific substrate degradation during the incubation. Upon digestion with Ty1 PR, the appearance of only a single fluorescent cleavage fragment was observed in the case of all types of cleavage sites (Fig 5). This implied that there are no alternative cleavage sites in the recombinant proteins and the substrates are cleaved only within the inserted Ty1 PR cleavage site sequences (Fig 5). To prove this, we performed analysis of cleavage fragments by MALDI-TOF MS, which is discussed later in *Identification of cleavage positions in the recombinant protein substrates* section.

The enzyme kinetic parameters of Ty1 PR were determined by fluorimetric assays. For the recombinant protein substrates containing a 10 residue-long cleavage site sequence the catalytic constants were found to have the same order of magnitude, and the highest value was observed for the substrate representing the PR/IN cleavage site when measured in cleavage buffer B (Table 1D). The catalytic efficiencies were higher for the substrates containing 20 residue-long cleavage site sequences than for those with shorter sequences (10 residue-long) (Table 1D). In contrast, the k_cat_/K_m_ constants were lower for the substrates containing longer IN/RT or Gag/PR cleavage site sequence if it was measured in cleavage buffer A (Table 1C), possibly due to the different buffer environments.

The importance of surface residues in substrate binding has recently been proven for HIV-1 PR. This binding surface has been referred as the substrate-groove [24]. The interdomain region between the matrix and capsid domains of HIV-1 polyprotein was found to contain ~20 residues, and is unstructured and accessible for the viral protease. In addition to the previously known S5-S5’ sites, HIV-1 PR was found to interact with those substrate residues of the interdomain linker which are not closed by the flaps (P12-P6 and P6’-P12’) (S1 Fig). The binding of additional residues along the cleavage position provides stronger interactions between the enzyme and the substrate compared to the shorter recognition sequences (P4-P4’) [24]. The comparison of catalytic constants measured for the substrates containing 10 and 20 residue-long cleavage site sequences was found to be insufficient to elucidate the presence of a substrate-groove in Ty1 PR. Therefore, in order to investigate whether Ty1 PR has a substrate-groove surface binding site similar to that of HIV-1 PR, a recombinant substrate containing a modified PR/IN cleavage site was also designed (PR/IN_20aa_mut). In this substrate the outer P10-P6 and P6’-P10’ cleavage site residues (PSNISVPTIN*NVHTSESTRK) were substituted to glycines (GGGGGVPTIN*NVHTSGGGGG) to disrupt all the possible side chain-mediated enzyme-substrate interactions at these sites. The k_cat_/K_m_ catalytic constants were comparable for PR/IN_20aa_wt and PR/IN_20aa_mut substrates (Table 1C and 1D). Glycine substitutions of the outer residues caused only slight changes of k_cat_/K_m_ values; the observed differences were found to be not statistically significant. While HIV-1 protease was found to have a functional substrate-groove being involved in substrate binding [24], our *in vitro* results imply that the contribution of the corresponding residues at the surface of Ty1 PR to the substrate binding may be negligible. The modification of P10-P6 and P6’-P10’ substrate residues—i.e. abolishment of side chain-side chain interactions at these sites—caused no significant changes in catalytic constants in any of the studied buffers. Therefore, we propose that Ty1 PR surface residues may have only weak interaction with the substrate at these sites.

### Identification of cleavage positions in the recombinant protein substrates

We found previously that the separation of cleavage products by SDS-PAGE may indicate the presence of alternative cleavage sites in the recombinant substrates [31], but the control cleavage site of TEV PR in a His_6_-MBP-mTurquoise2 fusion protein has not been used up to now in order to determine cleavage position of the studied protease.

Here we aimed to prove the lack of alternative cleavage positions; thus, the recombinant substrates were digested by Ty1 and TEV PRs, as well. The released short proteolytic fragments were then identified by MALDI-TOF MS, in the case of all studied substrate variants. Cleavage reactions by Ty1 PR were performed in cleavage buffer B, therefore, buffer exchange was performed (to 50 mM Tris, pH 8.0) in order to eliminate Tween20 buffer component which may interfere with MALDI-TOF MS analyses. We found that elimination of Tween20 by centrifugal filter units was successful, and that polyethylene glycol-derivatives did not impair detection of small proteolytic fragments.

After Ty1 and TEV PR digestion, the molecular masses of proteolytic fragments have been determined by MALDI-TOF MS and then were compared to the calculated m/z values ([M+H]^+^). The measured values corresponded well to the calculated ones (Fig 5). Results of MALDI-TOF MS were in agreement with those of SDS-PAGE analysis, and showed that the recombinant substrates are cleaved only at the desired positions by Ty1 PR. By these results we have proved that the protein substrates do not contain any alternative cleavage sites and are not cleaved by Ty1 PR neither at the inserted (GGGGS)_4_ flexible linker nor at the harbouring sequences.

### Inhibition studies

**Fig 6:**
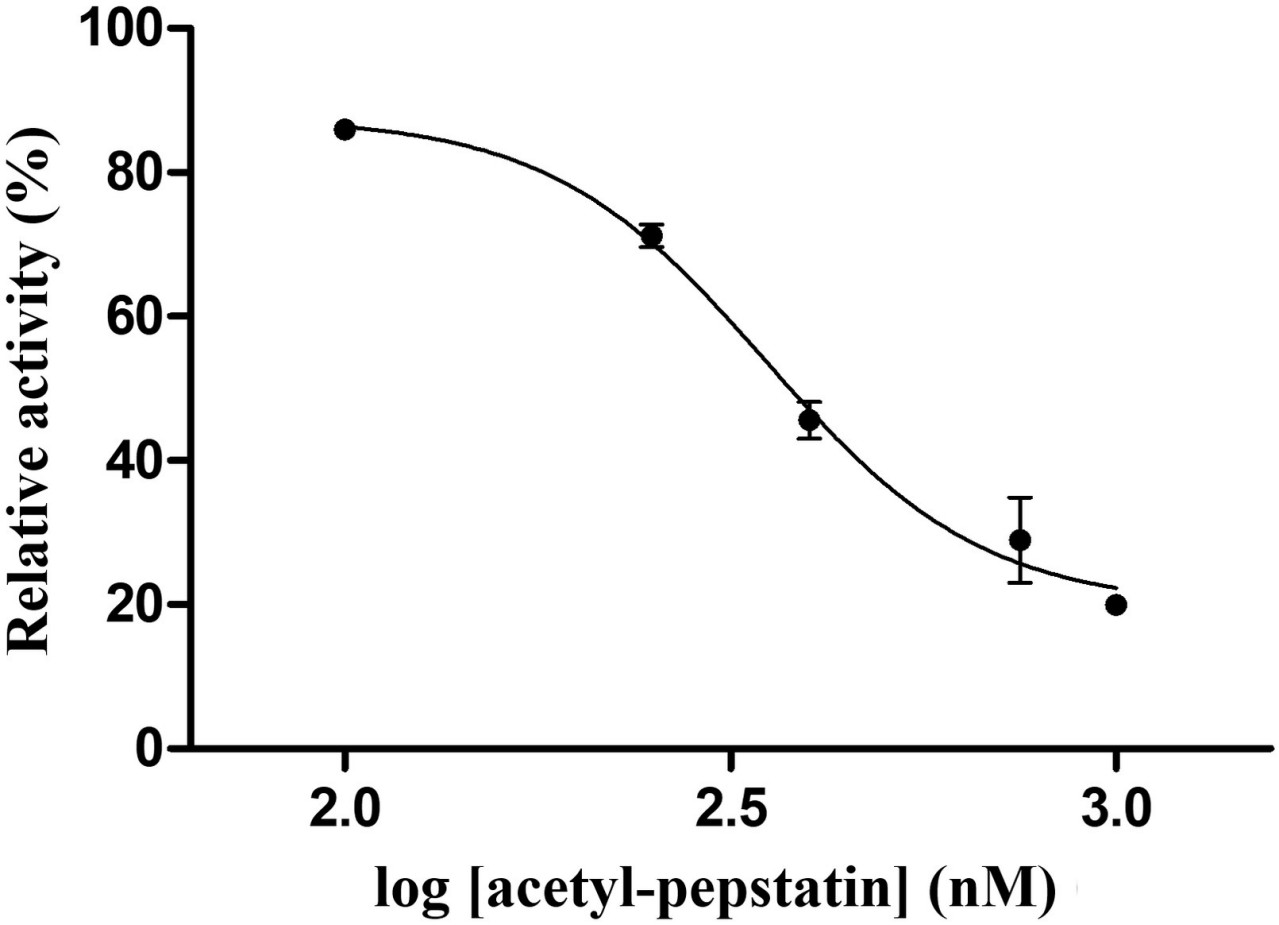
Inhibition of Ty1 PR-His_6_ by acetyl-pepstatin. Inhibitory constant was determined using VPTIN*NVHTS synthetic oligopeptide substrate. Activity measured in the absence of inhibitor was defined as 100%. Error bars represent SD (n = 3).

To test whether Ty1 protease is sensitive towards protease inhibitors, activity of Ty1 PR-His_6_ was measured in the presence of different inhibitors (Fig 6). Atazanavir, nelfinavir, saquinavir, darunavir, amprenavir, lopinavir, and tipranavir inhibitors have been approved by the Food and Drug Administration (FDA) and are applied in antiretroviral therapy, DMP-323 is a tight-binding inhibitor of HIV-1 PR, while acetyl-pepstatin and pepstatin A are classical inhibitors of aspartic proteases.

We found that only acetyl-pepstatin inhibited the proteolytic activity of Ty1 PR, other inhibitors were showed no inhibitory potential (at ≥100 nM final concentration). While amprenavir, atazanavir, darunavir, tipranavir, lopinavir, and DMP-323 have been reported to be able to inhibit XMRV PR [45], here we found that these molecules—as well as nelfinavir and saquinavir—were also unable to inhibit Ty1 PR. Both acetyl-pepstatin and pepstatin A were reported to be weak inhibitors of XMRV PR [29], and in our experiments pepstatin A showed no inhibitory potential on Ty1 PR-His_6_.

For acetyl-pepstatin, we determined IC_50_ as 367.5 nM and K_i_ as 296 nM (Fig 6). These results indicate that acetyl-pepstatin has a lower inhibitory potential for Ty1 PR than for HIV-1 PR (*K*_i_ = 13.15 nM, IC_50_ = 1.18 nM), but this value is more comparable with that one determined for XMRV PR (*K*_i_ = 712 nM, IC_50_ = 1290.2 nM) [29]. Interestingly, pepstatin A was found to be a potent inhibitor of the Copia transposon protease (*K*_i_ = 15 nM), with the sensitivity of the protease against pepstatin A closely resembling that of HIV-1 PR [17]. Despite the fact that out of the tested inhibitors only acetyl-pepstatin inhibited the enzyme activity, it should be considered only as a weak inhibitor of Ty1 PR. Interestingly, acetyl-pepstatin was found previously to have a unique binding mode to XMRV PR, and simultaneously two molecules can bind to the active site in a head-to-head orientation [29]. Future crystallographic studies may help to investigate whether the binding mode in the case of Ty1 PR resembles that of the XMRV-acetyl-pepstatin complex.

### *In silico* structural analysis

A proposed model was prepared for Ty1 protease by homology modeling because the structure of the protease has not been solved experimentally to date.

**Fig 7:**
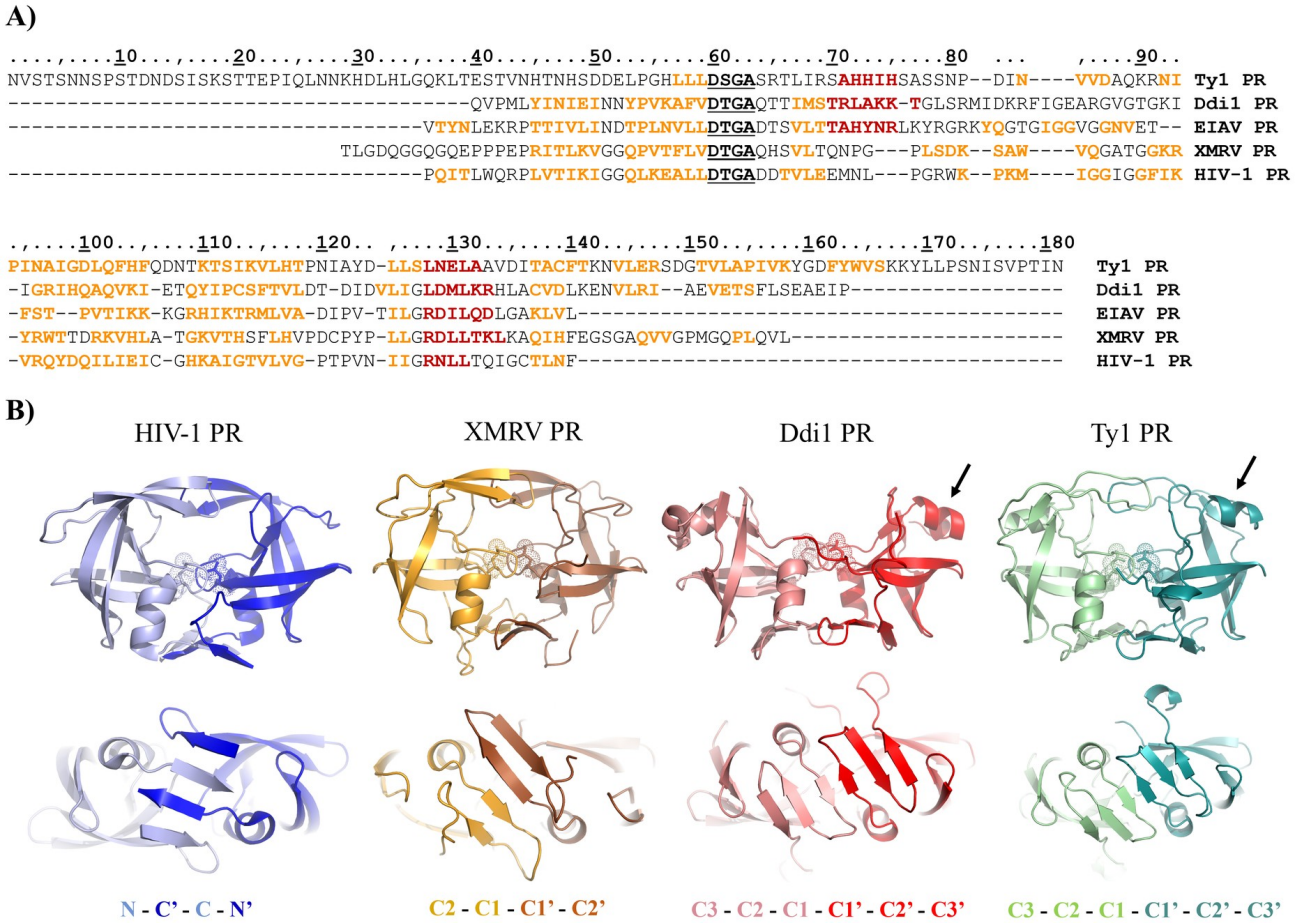
Sequences and structures of Ty1 PR and representative retroviral and retroviral-like proteases. **(A)** Sequences of Ty1, DNA damage-inducible protein 1 (Ddi1), equine infectious anemia virus (EIAV), xenotropic murine leukemia virus-related virus (XMRV), and human immunodeficiency virus type 1 (HIV-1) proteases were aligned. Arrangement of secondary structural elements is shown based on prediction for Ty1 PR, and based on crystal structures of Ddi1, EIAV, XMRV, and HIV-1 PRs, using DSSP (dictionary of protein secondary structure) images available in Protein Data Bank. Sequence numbering is shown for Ty1 PR. β-sheets and α-helices are indicated by orange and red, respectively. D-S/T-G-A catalytic motif residues are bold and underlined. **(B)** Cartoon representations are shown based on crystal structures of HIV-1, XMRV, and Ddi1 PRs, and based on homology model structure of Ty1 PR (41–164 residues). Upper panel shows the front views of the proteases, whereas bottom panel shows the enlarged views of dimer interfaces, together with the organizations of β-sheets. Additional helical inserts in the proximities of flaps (shown by arrows) are present only in the case of Ddi1 and Ty1 PRs. Catalytic aspartates are shown sticks and dots, while the monomers are differentiated by lighter and darker shades. N- and C-terminal extensions are not shown for Ty1 PR, structures of the full-length protease are shown in S3 Fig.

Based on the boundaries previously determined [20, 21], the protease domain of Ty1 is unusually long, consisting of 181 residues. It contains extended N- and C-terminal regions, which is not characteristic for retroviral and retroviral-like proteases (Fig 7A). To the best of our knowledge, neither the structural nor the functional roles of these extended regions have been explored to date. Notably, the presence of extensions has been observed in retroviral proteases. For example, both the N- and C-terminal regions of Moloney murine leukemia virus (Mo-MuLV) protease were found to be several residues longer than that of HIV-1 PR [46]; however, the N-terminal extension is considerably shorter than that of Ty1 PR. Although the presence of this extension shows no effect on the proteolytic activity of Mo-MuLV PR, precise processing of HIV-1 protease via cleavage of the N-terminal sequence (prior to the region being part of dimer interface) leads to increase of enzymatic activity [47]. While proteases of murine leukemia virus and XMRV show 98% sequence identity and differ only in two residues [29], both can be used for comparison with Ty1 PR, thus in Fig 7 we represent sequence of XMRV PR.

The *in silico* predictions showed a good agreement with the overall arrangement of the secondary structural elements with that of other retroviral and retroviral-like proteases (Fig 7A). Furthermore, the D-S-G-A sequence in Ty1 PR corresponds to the consensus D-S/T-G-A active-site motif of retroviral proteases, and Ty1 PR was predicted to share its general fold with the retroviral proteases (Fig 7B).

Predictions showed lack of ordered secondary structural elements (α-helices or β-strands) in the N-terminal region (N1-H56) of the protease. Disorder prediction also indicated the unstructured nature of this N-terminal extension, similarly to the extension in Mo-MuLV PR which was proposed to be flexible and has unknown conformation [46]. An α-helix was predicted to be possibly located near the catalytic motif of the protease (A71-H75), which may correspond to the additional helical insert previously observed for the Ddi1 [36] and equine infectious anemia virus (EIAV) proteases [48] (Fig 7A).

The results of predictions implied that the dimer interface of the homodimeric Ty1 PR contains only C-terminal β-sheets which are connected by short loops. In contrast with HIV-1 and equine infectious anemia virus (EIAV) proteases, Ty1 PR’s dimer interface consists of only C-terminal β-sheets which show no alternation. The C-terminal region of each monomer was predicted to contain four β-sheets (Fig 7A); however, none of the known retroviral or retroviral-like homodimeric aspartic proteases have eight-stranded dimeric interfaces. Therefore, we propose that homodimeric Ty1 PR’s dimer interface also consists of only six β-sheets (Fig 7B), similar to Ddi1 proteins [36]. As a consequence, the yeast Ddi1 protease structure was used to model the Ty1 PR dimer interface. Furthermore, without a proper template having an eight-stranded dimer interface, it was not possible to build a reliable eight-stranded interface model; exploration of the possible involvement of a fourth β-sheets in the dimer formation could only be estimated.

The higher sensitivity of Ty1 PR to urea, as compared to HIV-1 PR, can be explained by the differences in the organizations of dimer interfaces. While β-sheets of the N- and C-terminal regions alternate in the HIV-1 PR (Fig 7B), dimer interfaces of Ty1 and XMRV PRs comprise only C-terminal β-sheets showing no alternation (Fig 7B). Both XMRV [29] and Ty1 PRs showed lower dimer stabilities (e.g. higher sensitivity towards urea) compared to HIV-1. This implies that a dimer interface containing only C-terminal β-sheets without alternation provide lower stability for the homodimeric enzymes (e.g. for Ty1 and XMRV PRs), while alternating β-sheets ensure stronger interaction between the monomers (e.g. for HIV-1 PR).

Additionally, the sequence of the catalytic motif may also be a determinant of dimer stability. Homodimers of retroviral aspartic proteases are stabilized by intermonomeric interactions of Ser or Thr residues of the D-S/T-G-A consensus active site motif called “fireman’s grip”. It was found previously that the enzymes containing Ser in this motif instead of Thr may form less stable dimers: while T26S mutant HIV-1 protease exhibited lower specific activity compared to the wild-type [49], the S25T mutant HFV PR showed decreased sensitivity against urea [50]. Ty1 PR also contains Ser in this position (S2 Fig), which may also contribute to its lower dimer stability.

Structure of the full-length Ty1 PR was also modeled in order to investigate the extended N- and C-terminal regions (S3 Fig). These long regions are not present in the available structures of related aspartic proteases, therefore, without a template the predicted conformations of the extended regions were considered as highly approximate. Despite the poor model qualities of the N- and C-terminal regions, the proposed models of the full-length protease were used to support the interpretation of *in vitro* results. To study the putative involvement of substrate-groove residues in substrate binding, enzyme kinetic measurements were performed by recombinant protein substrates, but the recognition of P10-P6 and P6’-P10’ cleavage site residues by the Ty1 PR has not been established undoubtedly (Table 1D). The structures of the possible conformational variants (S3 Fig) implied that the surface residues of Ty1 PR may be not as accessible as in the substrate-groove of HIV-1 PR, due to the putative proximities of the N- and C-terminal extensions to the active site and enzyme surface. Considering this potential steric hindrance to substrate binding, we presumed that the binding surface for P10-P6 and P6’-P10’ substrate residues in Ty1 PR is absent or has a different structure than that of the substrate-groove of HIV-1 protease [24]. Without having more accurate model complexes or performing extended molecular dynamical calculations, we were unable to explore either whether the surface residues of Ty1 PR are accessible for recognition or the mechanism underlying the involvement of long N- and C-terminal regions in substrate binding.

Natural cleavage site sequence of Ty1 and Ty3 proteases have already been analyzed and average hydrophobicity indexes were determined for P10-P10’ residues of Ty PR cleavage sites [20]. The comparison revealed remarkable differences of specificities compared to retroviral protease cleavage sites, but protease structures have not been compared previously. Here we used the proposed model structure of Ty1 PR to study substrate binding cavities. The amino acid compositions of P4-P1 sites were determined by structure-based alignment of HIV-1 and Ty1 PRs. The cavity compositions have already been determined for HIV-1 PR [51, 52], the substrate binding cavities of Ty1 PR have been mapped by the identification of residues in the corresponding positions (S4 Fig).

We found that the S1 binding site of Ty1 PR consists of mainly hydrophobic residues, and thus is similar to the S1 site of HIV-1 PR. However, while retroviral proteases prefer binding of predominantly hydrophobic P1 residues [52], based on average hydrophobicities of all three known Ty1 cleavage sites both the P1 and P1’ residues are hydrophilic [20]. S2 site is also hydrophobic based on the model structure, in agreement with the higher hydrophobicity of P2 and P2’ residues, which are mainly Val or Ile in the cleavage site sequences (S4 Fig). Most of the residues forming the S3 site were found to be hydrophilic, in agreement with the cleavage site sequences which contain almost exclusively hydrophilic residues in P3 and P3’ positions. Based on average values the P4 and P4’ residues are not highly hydrophobic or hydrophilic, and the S4 site was found to be comprised by mainly hydrophobic residues; however, this site is less well-defined and is exposed to the surface [51]. The average distribution of hydrophobic, hydrophilic and charged residues in the substrate binding cavities showed no significant differences compared to HIV-1 PR, with the exception of S3 site of Ty1 PR which contains no charged residues. While the binding site compositions are mostly in agreement with the hydrophobicity profiles of cleavage site sequences, the specificities cannot be estimated accurately purely based on binding cavity compositions. Extended *in silico* calculations on enzyme-ligand complexes and *in vitro* enzymatic experiments using a series of modified substrates would be necessary for the detailed investigation of enzyme specificity, which was out of the scope of this study.

## Discussion

In this study we performed the biochemical characterization of recombinant Ty1 PR expressed in bacterial cells. Both untagged and His_6_-tagged forms of the enzyme were expressed. While untagged Ty1 PR was purified by gel filtration, affinity chromatography was used to purify Ty1 Gag-PR-His_6_ recombinant protein. In agreement with the known autoproteolysis of the Gag protein [20], we also observed self-processing of Ty1 Gag-PR-His_6_ precursor, and in the activity assays we used the processed Ty1 PR-His_6_ form of the enzyme.

In order to investigate the biochemical characteristics of Ty1 PR, activity measurements have been performed. Highest activities were measured at higher salt concentrations (> 1 M NaCl), and the slightly alkaline pH and 30°C temperature were optimal for the enzyme, suggesting a general adaptation to the intracellular life-cycle and lower temperature optimum for yeasts. Additionally, the observed temperature sensitivity of protease may contribute to that of Ty1 transposition efficiency, as well. While the optimal temperature for the protease was found to be close to 30 °C, this temperature is known to be suboptimal for the transposition of most yeast strains, which showed highest transposition efficiency at ~24 °C [23]. Not only the protease, but the reverse transcriptase is also a determinant of the temperature sensitivity of transposition, in virus-like particles formed at 37 °C the RT activity was severely impaired in case of Ty1 [23]. Temperature-induced conformational changes of the template/primer complex and Gag-Pol polyprotein were considered to contribute to temperature sensitivity of Ty1. In contrast with the previously observed insensitivity of exogenous Ty1 RT on temperature *in vitro* [23], our results imply that Ty1 PR is inherently temperature sensitive and therefore it may contribute to the temperature-dependence of transposition efficiency. The urea concentration leading to 50% loss in enzymatic activity was found to be substantially lower than in the case of HIV-1 PR, and was more similar to that of XMRV PR [29]. Proteolytic assays showed very low specific activity of Ty1 PR compared to retroviral proteases; the obtained values were comparable with that of HFV and AMV PRs. The sensitivity of Ty1 retrotransposon PR against protease inhibitors has not been tested so far. We found that all tested protease inhibitors—which have been designed against HIV-1 PR and are applied in antiretroviral therapies—were inefficient against Ty1 PR. Only a general aspartic protease inhibitor acetyl-pepstatin showed inhibitory potential, while pepstatin A was unable for the inhibition of Ty1 PR.

Neither experimental nor in silico methods have been applied to investigate the structural characteristics of Ty1 PR until now. We predicted both secondary and quaternary structure of Ty1 PR by homology modeling. The protease was found to share its overall fold and the conserved active site motif with HIV-1 PR, but some structural characteristic differ from that of retroviral proteases. Due to the putative presence of an additional helical insert and the N- and C-terminal extended regions, Ty1 PR shows higher structural similarity with the retroviral-like Ddi1 proteases rather than with HIV-1 PR. Furthermore, dimer interface organization of Ty1 PR was predicted to be more similar to that of XMRV and Ddi1 PRs. Consistent with the results of *in vitro* stability analyses, dimer interfaces consisting of non-alternating C-terminal β-sheets provide only lower dimer stability and higher sensitivity to urea, as we observed it for Ty1 PR and has previously been reported for XMRV PR [29]. The sequence of the D-S/T-G-A motif may also contribute to lower dimer stability, because a Ser residue in the catalytic motif can provide lower stability in the “fireman’s grip” compared to a Thr [49].

Besides the synthetic oligonucleotides widely used in protease assays, fluorescent protein-containing substrates were also used in activity measurements. The applied fluorimetric protease assay has been designed and tested previously on HIV-1 and TEV PRs [31–33], and we successfully adapted it for the investigation of Ty1 PR. The previously designed pDest-His_6_-MBP-mTurquoise2 expression vector [31–33] has been modified to contain the coding sequence of a (GGGGS)_4_ linker. In the recombinant substrate, this flexible linker was expected to provide flexibility for the fluorescent tag and accessibility for the cleavages site. The prepared protein substrates contained different cleavage site sequences of Ty1 PR, the sequences of which having been determined previously [20]. We used SDS-PAGE and MALDI-TOF MS analyses to prove that Ty1 PR cleaves the protein substrates only at the desired positions within the incorporated target sequences. Substrates containing wild-type or modified cleavage site sequences were also designed and have been used to investigate the putative presence of a substrate-binding surface (corresponding to substrate-groove of HIV-1) in Ty1 PR. Our results did not find evidence for the presence of such a substrate-groove in Ty1 PR. Based on the proposed model structures, the residues building the S4-S1 substrate binding cavities have been identified and we infer that interactions with P10-P6 and P6’-P10’ residues in the case of Ty1 PR differ compared to HIV-1 PR. The effect of the extended N- and C-terminal regions of Ty1 PR on substrate binding or on the accessibility of the enzyme surface for substrate binding remains unclear.

## Supporting information

S1 Table List of applied oligonucleotide primers. The primer sequences listed in this table have been deposited into the public oligonucleotide database of the Laboratory of Retroviral Biochemistry (http://lrb.med.unideb.hu/index.php/research/oligos).
(DOCX)

S1 Fig Representation of the substrate groove of HIV-1 PR. (A) Modeled complex of HIV-1 PR with a peptide substrate representing P5-P4 residues of HIV-1 matrix/capsid cleavage site. The peptide residues are bound to the active site of the enzyme (S5-S4’ binding sites). The model complex was prepared by the method described previously [51]. (B) Modeled complex of HIV-1 PR with a peptide substrate representing P12-P12’ residues of the same cleavage site. While P5-P5’ residues are bound to the active site, the P12-P6 and P6’-P12’ residues interact with the S-groove at the enzyme surface. The modeled complex was prepared and kindly provided by Gary S. Laco [24], the figure was prepared without modification of the original coordinates. The protease is shown by surface representation, while the peptide by sticks, sequences of the substrates are also indicated.
(TIF)

S2 Fig Fireman’s grip in Ty1 PR. (A) Side view of the homology model of homodimeric Ty1 PR. The monomers are colored by different shades, catalytic aspartates are also shown in the active site (boxed). (B) The active site is highlighted, residues are shown in top view. Hydrogen bonds around the catalytic aspartates are shown by grey dotted lines, distances are also indicated (Å).
(TIF)

S3 Fig Ty1 PR contains N- and C-terminal extensions. (A) Result of secondary structure prediction for the full-length Ty1 PR is shown based on Fig 7A. β-sheets are colored by orange, while α-helices are red, the residues of the catalytic motif are bold and underlined. (B) The proposed model of homodimeric Ty1 PR (41–164 residues) of the protease modeled without the extensions is shown without the terminal extensions. (C-D) The front (C) and top views (D) of superimposed models containing both N- and C-terminal extensions (1–40 and 156–181 residues, respectively) are also represented, the extensions are shown by different colors.
(TIF)

S4 Fig Compositions of S4-S1 substrate binding cavities in HIV-1 and Ty1 PRs. (A) Substrate binding site compositions of HIV-1 PR were determined previously [51, 52], while the residues of Ty1 PR in the corresponding positions based on structure-based alignment. Residues involved in putative side chain-side chain interactions are shown by bold letters, otherwise are shown in italics. (B) Average hydrophobicities of Ty1 PR cleavage site residues were determined based on the values described by Kyte and Doolittle [53] and are shown for P5-P5' positions. Red arrow shown cleavage position.
(TIF)

S1 Raw Images (PDF)
