# Supplementary material for: Biochemical characterization of Ty1 retrotransposon protease
Source: PLoS One. 2020 Jan 9;15(1):e0227062. doi: 10.1371/journal.pone.0227062 (PMC6952103; doi:10.1371/journal.pone.0227062)
Supplement: S1 Table — The primer sequences listed in this table have been deposited into the public oligonucleotide database of the Laboratory of Retroviral Biochemistry (http://lrb.med.unideb.hu/index.php/research/oligos). (DOCX) [file pone.0227062.s001.docx]

**S1 Table. List of applied oligonucleotide primers.** The primer sequences listed in this table have been deposited into the public oligonucleotide database of the Laboratory of Retroviral Biochemistry (<http://lrb.med.unideb.hu/index.php/research/oligos>).

| **Name** | **Sequence** |
| --- | --- |
| **Ty1 PR forward** | 5'-GCGCATATGAATGTATCCACATCTAATAACTCTCC-3 |
| **Ty1 PR reverse** | 5'-CGCGGATCCATTGATGGTGGGTACGG-3' |
| **New-cloning cassette forward** | 5’TAATCTTCTGGATCCGGTGGCGGCGGCTCAGGTGGTGGTGGCTCGGGTGGCGGCGGTTCGGGCGGTGGTGGCTCAG-3’ |
| **New-cloning cassette reverse** | 5’CTAGCTGAGCCACCACCGCCCGAACCGCCGCCACCCGAGCCACCACCACCTGAGCCGCCGCCACCGGATCCAGAAGATTAAT-3’ |
| **PR/IN 10aa wt forward** | 5’- TAAAgtacccaccatcaataatgtccatacaagtG - 3’ |
| **PR/IN 10aa wt reverse** | 5’- GATCCACTTGTATGGACATTATTGATGGTGGGTACTTTAAT - 3’ |
| **PR/IN 20aa wt forward** | 5’- TAAACCATCAAATATCTCCGTACCCACCATCAATAATGTCCATACAAGTGAAAGT ACACGCAAAG - 3’ |
| **PR/IN 20aa wt reverse** | 5’-GATCCTTTGCGTGTACTTTCACTTGTATGGACATTATTGATGGTGGGTACGGAGATATTTGATGGTTTAAT - 3’ |
| **PR/IN 20aa mut forward** | 5’- TAAAggaggcgggggtggagtacccaccatcaataatgtccatacaagtggaggc gggggtggaG - 3’ |
| **PR/IN 20aa mut reverse** | 5’ - GATCCTCCACCCCCGCCTCCACTTGTATGGACATTATTGATGGTGGGTACTCC ACCCCCGCCTCCTTTAAT - 3’ |
| **IN/RT 10aa wt forward** | 5’- TAAAattcacctgattgcagctgtaaaagcagtaG - 3’ |
| **IN/RT 10aa wt reverse** | 5’- GATCCtactgcttttacagctgcaatcaggtgaatTTTAAT - 3’ |
| **IN/RT 20aa wt forward** | 5’- TAAAagatcgaagaaacgaattcacctgattgcagctgtaaaagcagtaaaatcaatcaaaccaG - 3’ |
| **IN/RT 20aa wt reverse** | 5’- GATCCtggtttgattgattttactgcttttacagctgcaatcaggtgaattcgtttcttcgatctTTTAAT - 3’ |
| **Gag/PR 10aa wt forward** | 5’- TAAAacagccagggctcacaatgtatccacatctG - 3’ |
| **Gag/PR 10aa wt reverse** | 5’- GATCCagatgtggatacattgtgagccctggctgtTTTAAT - 3’ |
| **Gag/PR 20aa wt forward** | 5’-TAAAaattcgaaatcgaaaacagccagggctcacaatgtatccacatctaataactctcccagcG - 3’ |
| **Gag/PR 20aa wt reverse** | 5’- GATCCgctgggagagttattagatgtggatacattgtgagccctggctgttttcgatttcgaattTTTAAT - 3’ |
| **sequencing forward primer** | 5'-GATGAAGCCCTGAAAGACGCGCAG-3' |
