# Supplementary figures and images for: Biochemical characterization of Ty1 retrotransposon protease
Source: PLoS One. 2020 Jan 9;15(1):e0227062. doi: 10.1371/journal.pone.0227062 (PMC6952103; doi:10.1371/journal.pone.0227062)

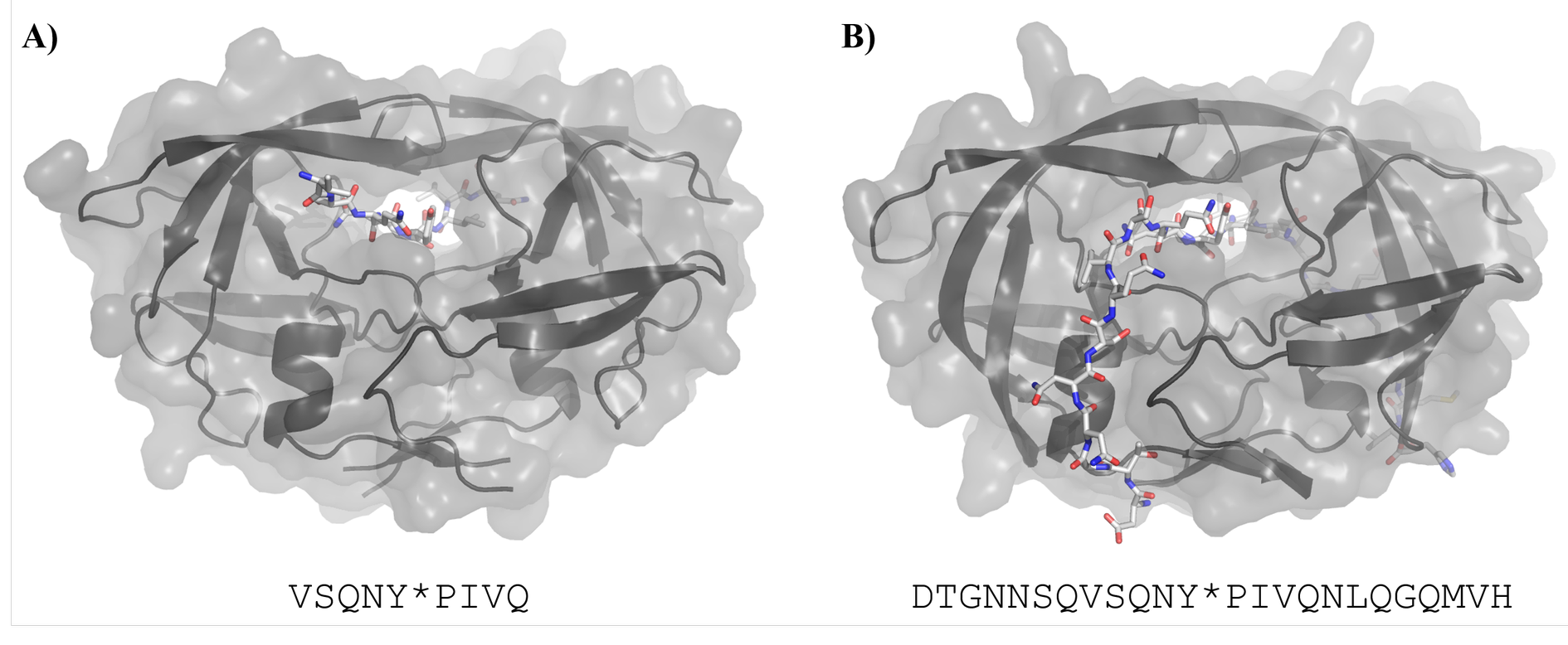

Supplement: S1 Fig — (A) Modeled complex of HIV-1 PR with a peptide substrate representing P5-P4 residues of HIV-1 matrix/capsid cleavage site. The peptide residues are bound to the active site of the enzyme (S5-S4’ binding sites). The model complex was prepared by the method described previously [51]. (B) Modeled complex of HIV-1 PR with a peptide substrate representing P12-P12’ residues of the same cleavage site. While P5-P5’ residues are bound to the active site, the P12-P6 and P6’-P12’ residues interact with the S-groove at the enzyme surface. The modeled complex was prepared and kindly provided by Gary S. Laco [24], the figure was prepared without modification of the original coordinates. The protease is shown by surface representation, while the peptide by sticks, sequences of the substrates are also indicated. (TIF) [file pone.0227062.s002.tif]

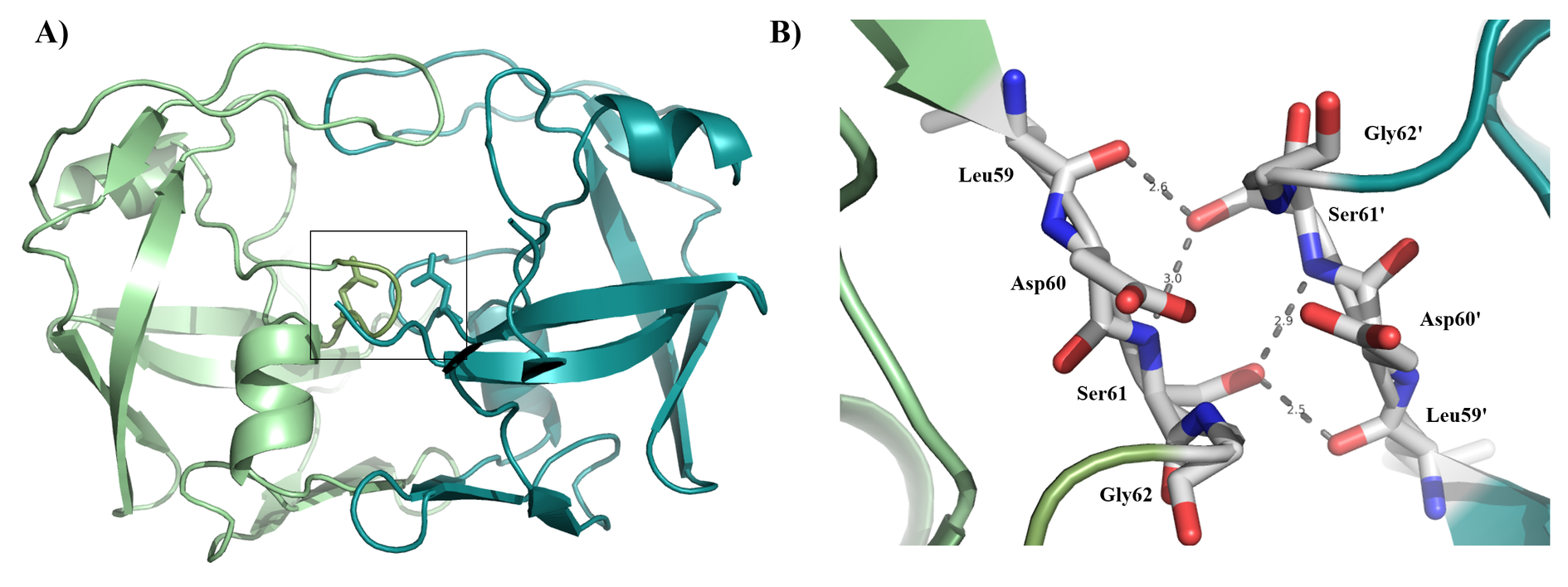

Supplement: S2 Fig — (A) Side view of the homology model of homodimeric Ty1 PR. The monomers are colored by different shades, catalytic aspartates are also shown in the active site (boxed). (B) The active site is highlighted, residues are shown in top view. Hydrogen bonds around the catalytic aspartates are shown by grey dotted lines, distances are also indicated (Å). (TIF) [file pone.0227062.s003.tif]

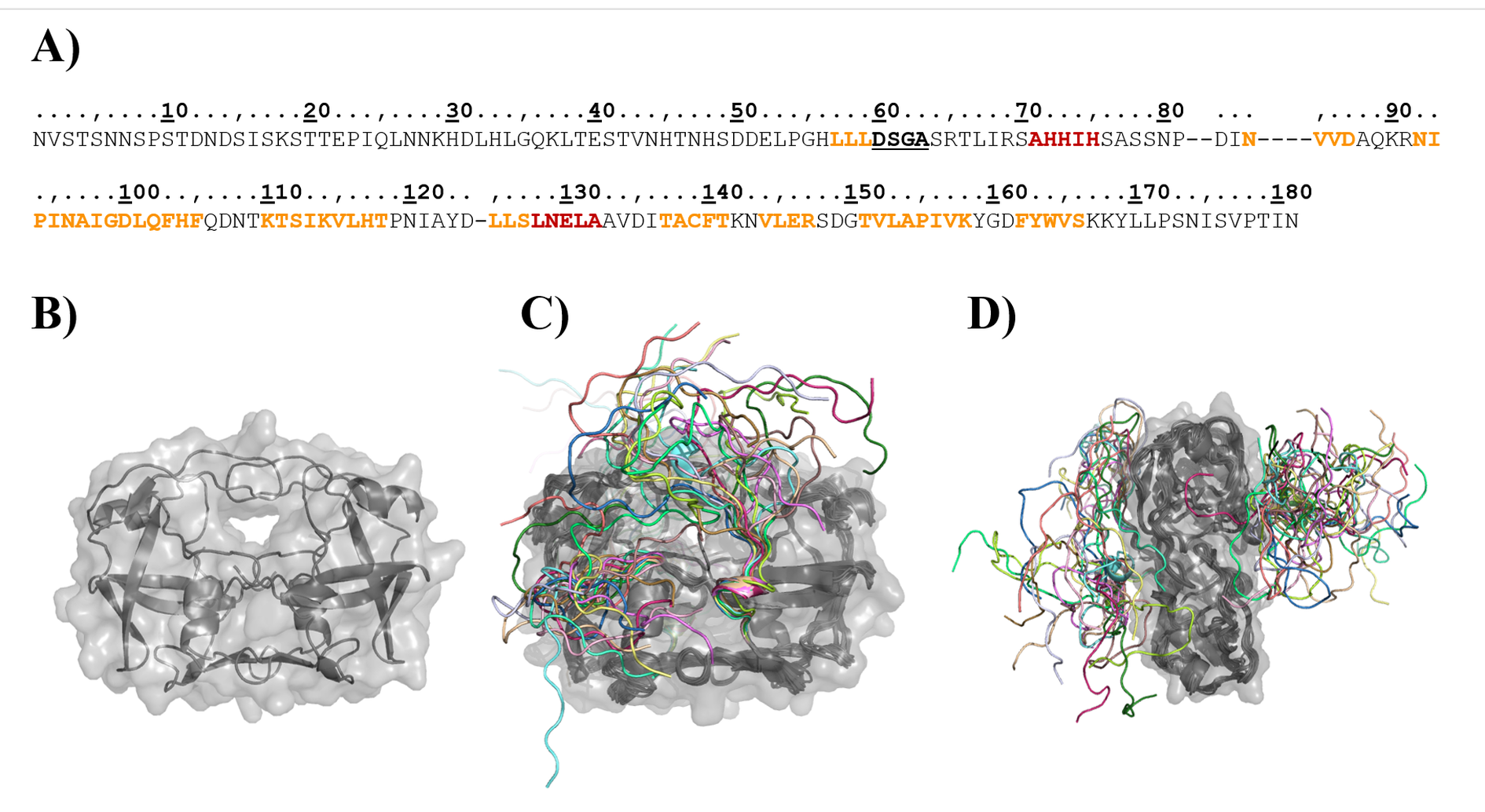

Supplement: S3 Fig — (A) Result of secondary structure prediction for the full-length Ty1 PR is shown based on Fig 7A. β-sheets are colored by orange, while α-helices are red, the residues of the catalytic motif are bold and underlined. (B) The proposed model of homodimeric Ty1 PR (41–164 residues) of the protease modeled without the extensions is shown without the terminal extensions. (C-D) The front (C) and top views (D) of superimposed models containing both N- and C-terminal extensions (1–40 and 156–181 residues, respectively) are also represented, the extensions are shown by different colors. (TIF) [file pone.0227062.s004.tif]

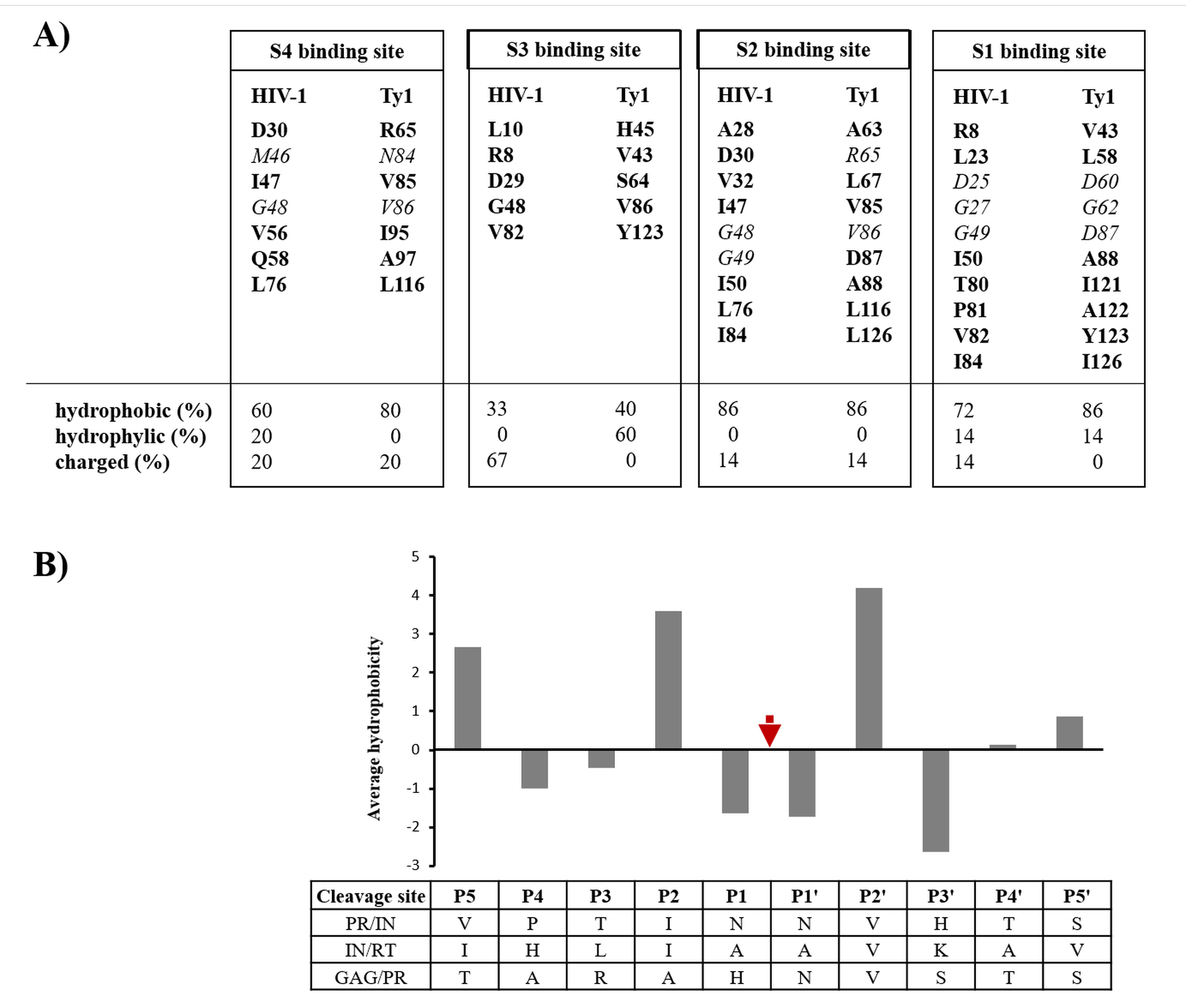

Supplement: S4 Fig — (A) Substrate binding site compositions of HIV-1 PR were determined previously [51, 52], while the residues of Ty1 PR in the corresponding positions based on structure-based alignment. Residues involved in putative side chain-side chain interactions are shown by bold letters, otherwise are shown in italics. (B) Average hydrophobicities of Ty1 PR cleavage site residues were determined based on the values described by Kyte and Doolittle [53] and are shown for P5-P5' positions. Red arrow shown cleavage position. (TIF) [file pone.0227062.s005.tif]
