# Supplementary material for: Biochemical characterization of Ty1 retrotransposon protease
Source: PLoS One. 2020 Jan 9;15(1):e0227062. doi: 10.1371/journal.pone.0227062 (PMC6952103; doi:10.1371/journal.pone.0227062)
Supplement: S1 Raw Images — (PDF) [file pone.0227062.s006.pdf]

**Figure 3A.**

Unedited original gel image. Image was taken by using AlphaImager HP system (Protein Simple, USA).

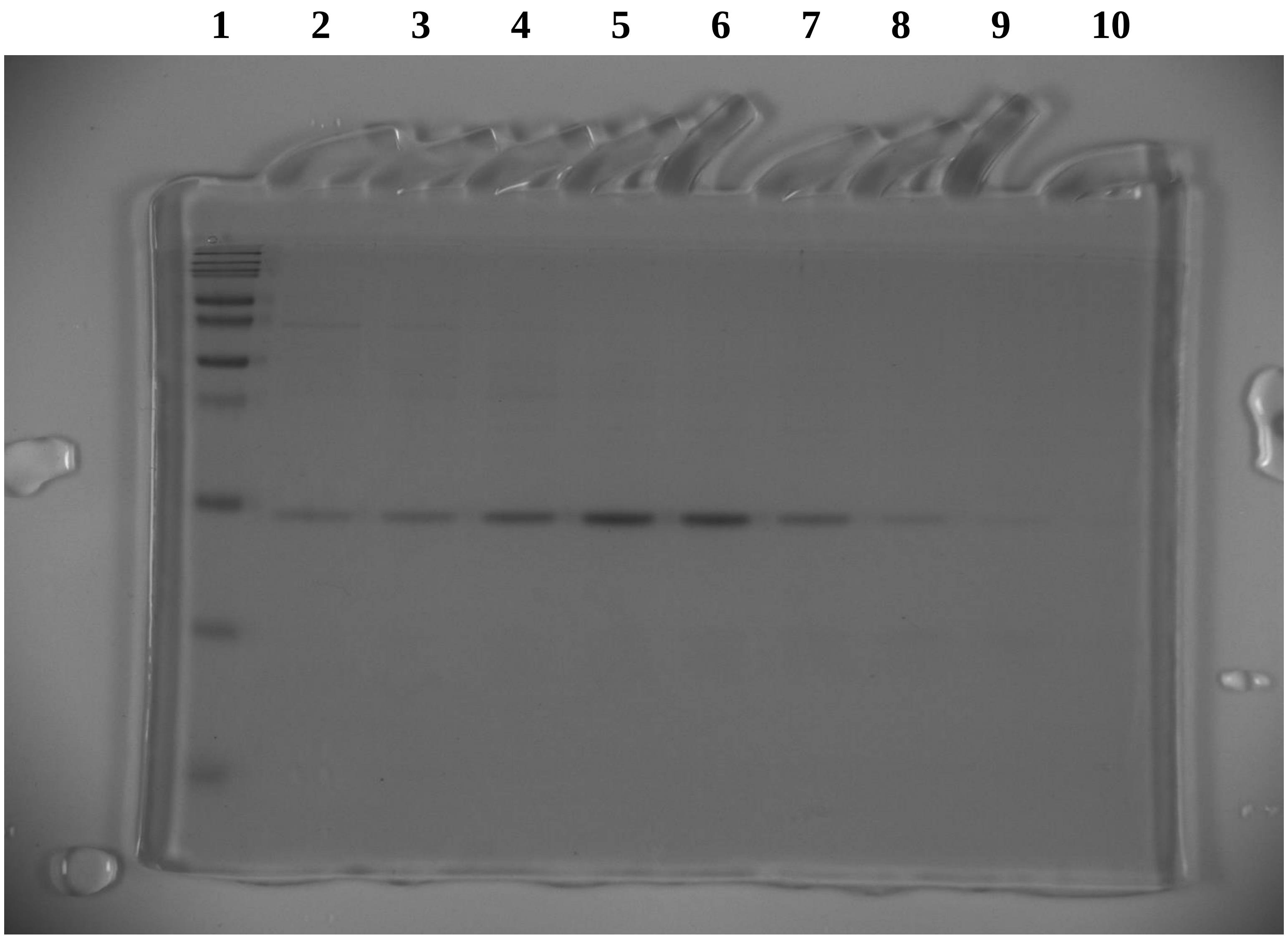


**Sample**

**1. Molecualr weight standard**
(Prosieve QuadColor)

**2. Eluate fraction I.**

**3. Eluate fraction II.**

**4. Eluate fraction III.**

**5. Eluate fraction IV.**

**6. Eluate fraction V.**

**7. Eluate fraction VI.**

**8. Eluate fraction VII.**

**9. Eluate fraction VIII.**

**10. Eluate fraction IX.**

**Figure 3B.**

Unedited original blot image. Image was taken by using AlphaImager HP system (Protein Simple, USA).

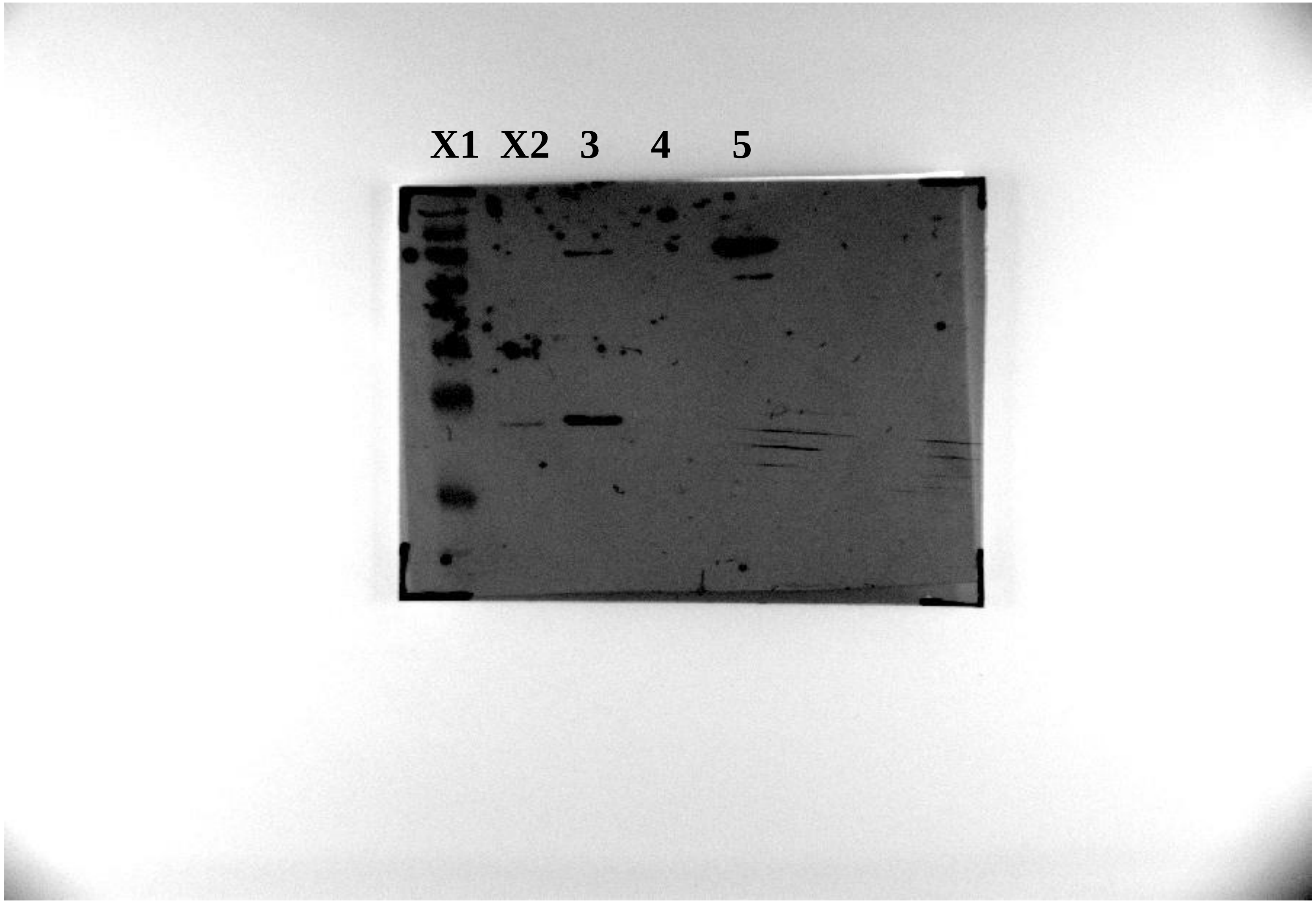


**Sample**

**X1. Molecualr weight standard**
(Prosieve Prestained Prot. Ladder Plus)

**X2. Eluate fraction I.**

**3. Eluate fraction II.**

**4. Eluate fraction III.**

**5. Flowthrough fraction I.**

**Figure 3C.**
Unedited original gel image. Image was taken by using AlphaImager HP system (Protein Simple, USA).

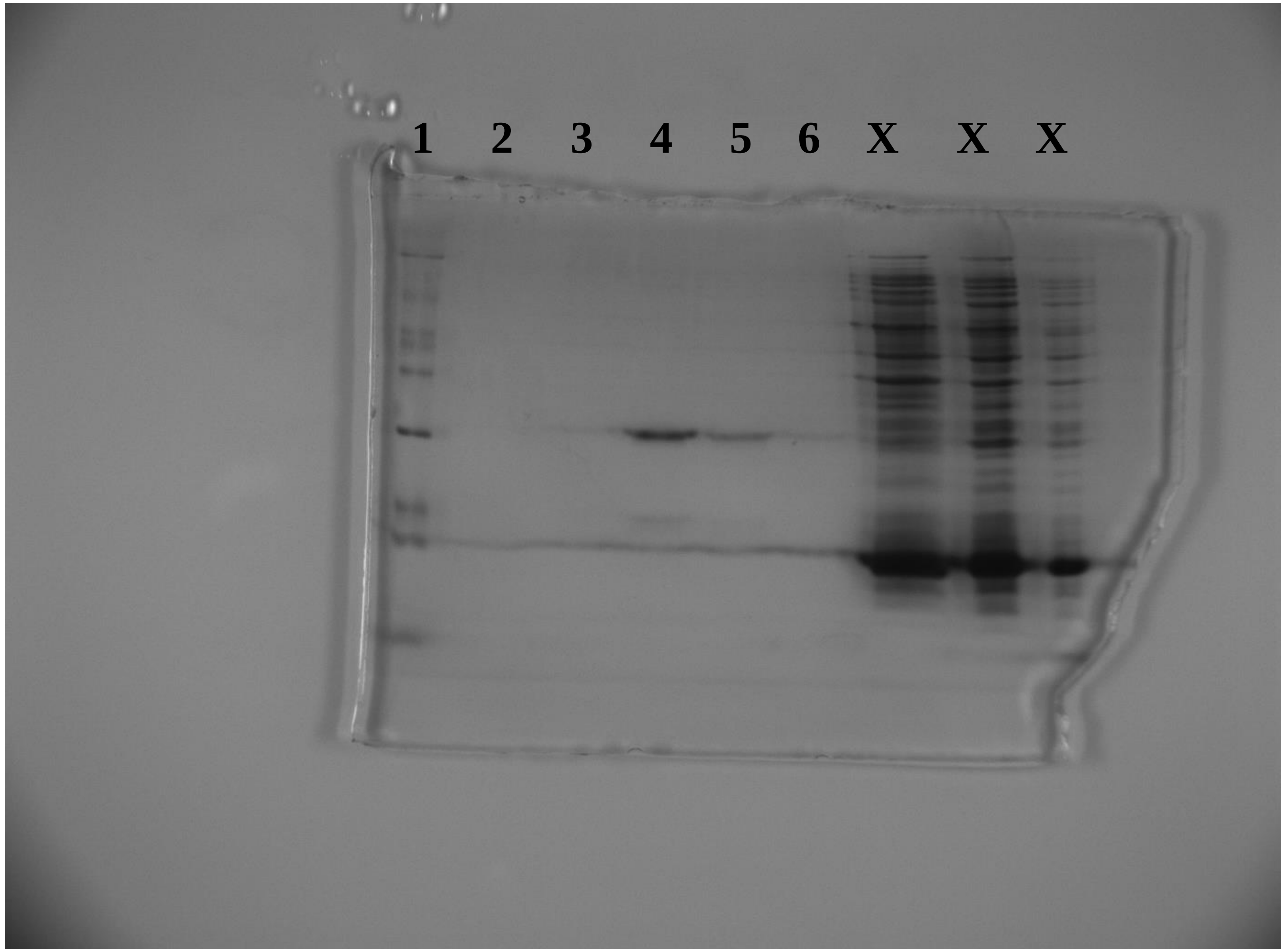


**Sample**

**1. Molecualr weight standard**
(Unstained Protein Ladder, SM0431)

**2. Eluate fraction I.**

**3. Eluate fraction II.**

**4. Eluate fraction III.**

**5. Eluate fraction IV.**

**6. Eluate fraction V.**

**Figure 5B.**

Unedited original gel image.

To prepare Figure 5B, bands were visualized in the same gel using three different methods. Fluorescence was detected by UV or Blue light transillumination, while image for Coomassie-stained gels were taken under visible light.

Here **Blue light transilluminator** was used for detection, the colored image was taken by a camera.

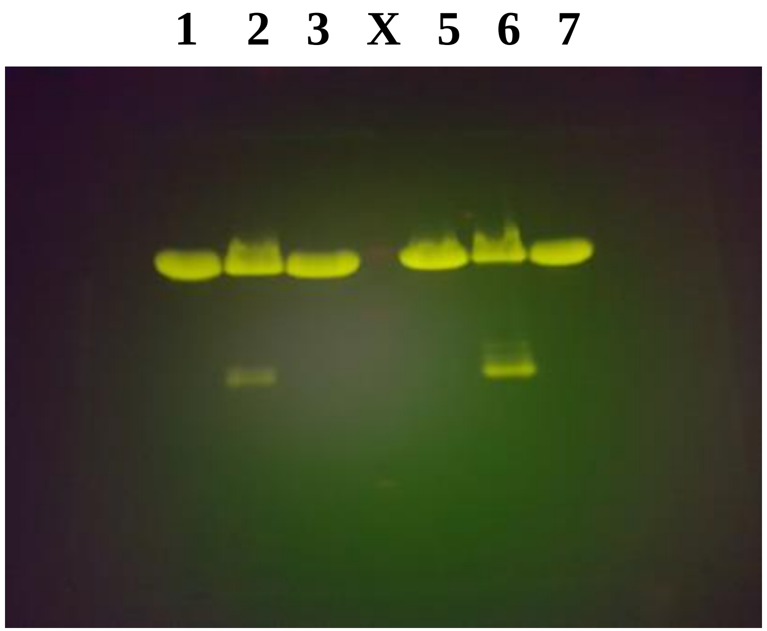


| | Substrate | Enzyme | Incubation (h) |
|---|---|---|---|
| **1.** | **PR/IN_10aa_wt** | **-** | **0** |
| **2.** | **PR/IN_10aa_wt** | **+** | **16** |
| **3.** | **PR/IN_10aa_wt** | **-** | **16** |

**X. Molecualr weight standard** (Prosieve QuadColor)

| | Substrate | Enzyme | Incubation (h) |
|---|---|---|---|
| **5.** | **PR/IN_20aa_wt** | **-** | **0** |
| **6.** | **PR/IN_20aa_wt** | **+** | **16** |
| **7.** | **PR/IN_20aa_wt** | **-** | **16** |

**Figure 5B.**

Unedited original gel image.

To prepare Figure 5B, bands were visualized in the same gel using three different methods. Fluorescence was detected by UV or Blue light transillumination, while image for Coomassie-stained gels were taken under visible light.

Here **Blue light transilluminator** was used for detection, the colored image was taken by a camera.

**1 2 3 X 5 6 7 X**

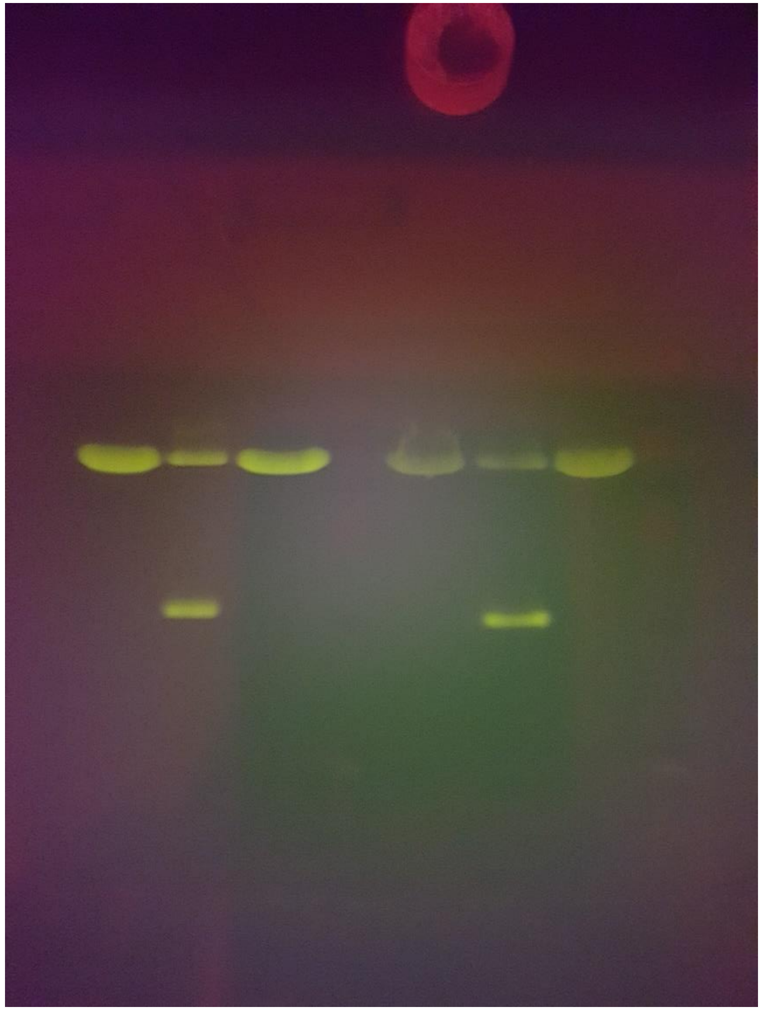

| | **Substrate** | **Enzyme** | **Incubation (h)** |
|---|---|---|---|
| **1.** | **GAG/PR_20aa_wt** | **-** | **0** |
| **2.** | **GAG/PR_20aa_wt** | **+** | **16** |
| **3.** | **GAG/PR_20aa_wt** | **-** | **16** |
| **X. Molecualr weight standard** (Prosieve QuadColor) | | | |
| **5.** | **IN/RT_20aa_wt** | **-** | **0** |
| **6.** | **IN/RT_20aa_wt** | **+** | **16** |
| **7.** | **IN/RT_20aa_wt** | **-** | **16** |
| **X. Molecualr weight standard** (Prosieve QuadColor) | | | |

**Figure 5B.**

Unedited original gel image.

To prepare Figure 5B, bands were visualized in the same gel using three different methods. Fluorescence was detected by UV or Blue light transillumination, while image for Coomassie-stained gels were taken under visible light.

Here **UV light** was used for detection, the image was taken by AlphaImager HP system (Protein Simple, USA).

**This image is not part of any figure in the manuscript.**

**1 2 3 X 5 6 7**

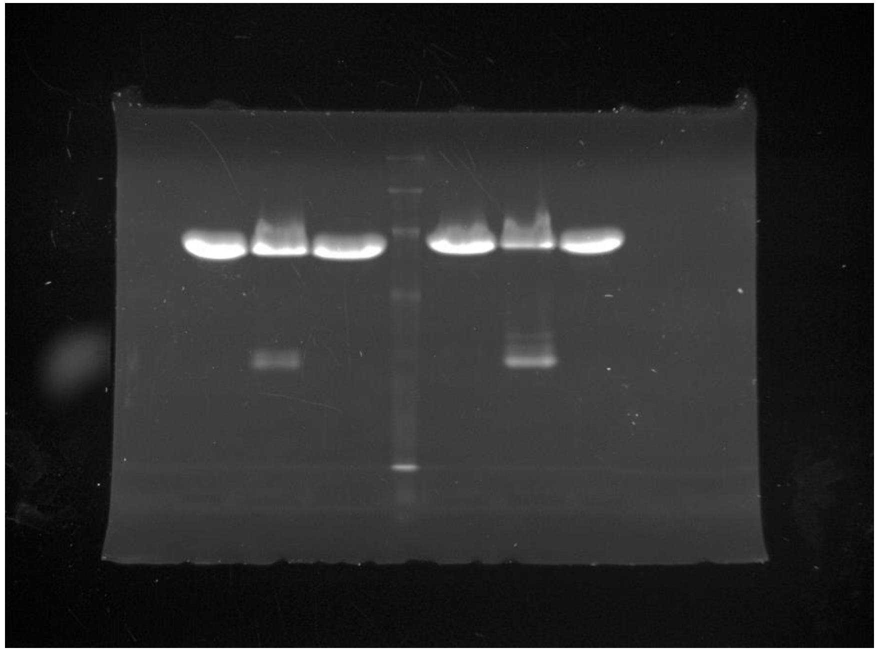

| | Substrate | Enzyme | Incubation (h) |
|---|---|---|---|
| **1.** | **PR/IN_10aa_wt** | **-** | **0** |
| **2.** | **PR/IN_10aa_wt** | **+** | **16** |
| **3.** | **PR/IN_10aa_wt** | **-** | **16** |

**X. Molecualr weight standard** (Prosieve QuadColor)

| | | | |
|---|---|---|---|
| **5.** | **PR/IN_20aa_wt** | **-** | **0** |
| **6.** | **PR/IN_20aa_wt** | **+** | **16** |
| **7.** | **PR/IN_20aa_wt** | **-** | **16** |

**Figure 5B.**

Unedited original gel image.

To prepare Figure 5B, bands were visualized in the same gel using three different methods. Fluorescence was detected by UV or Blue light transillumination, while image for Coomassie-stained gels were taken under visible light.

Here **UV light** was used for detection, the image was taken by AlphaImager HP system (Protein Simple, USA).

**This image is not part of any figure in the manuscript.**

**1 2 3 X 5 6 7 X**

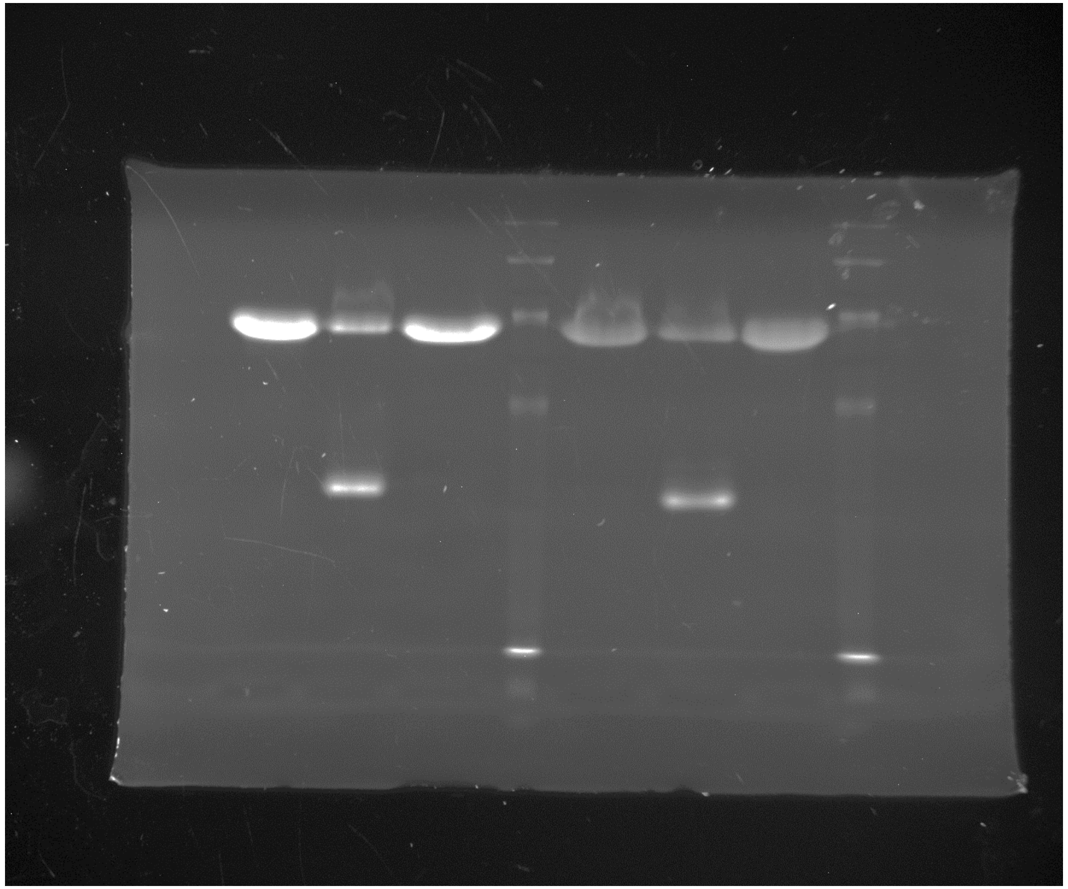

| | **Substrate** | **Enzyme** | **Incubation (h)** |
|---|---|---|---|
| **1.** | **GAG/PR_20aa_wt** | **-** | **0** |
| **2.** | **GAG/PR_20aa_wt** | **+** | **16** |
| **3.** | **GAG/PR_20aa_wt** | **-** | **16** |

**X. Molecualr weight standard** (Prosieve QuadColor)

| | | | |
|---|---|---|---|
| **5.** | **IN/RT_20aa_wt** | **-** | **0** |
| **6.** | **IN/RT_20aa_wt** | **+** | **16** |
| **7.** | **IN/RT_20aa_wt** | **-** | **16** |

**X. Molecualr weight standard** (Prosieve QuadColor)

**Figure 5B.**

Unedited original gel image.

To prepare Figure 5B, bands were visualized in the same gel using three different methods. Fluorescence was detected by UV or Blue light transillumination, while image for Coomassie-stained gels were taken under visible light.

Here a **Coomassie-stained** gel is shown, the image was taken by AlphaImager HP system (Protein Simple, USA).

**1 2 3 X 5 6 7**

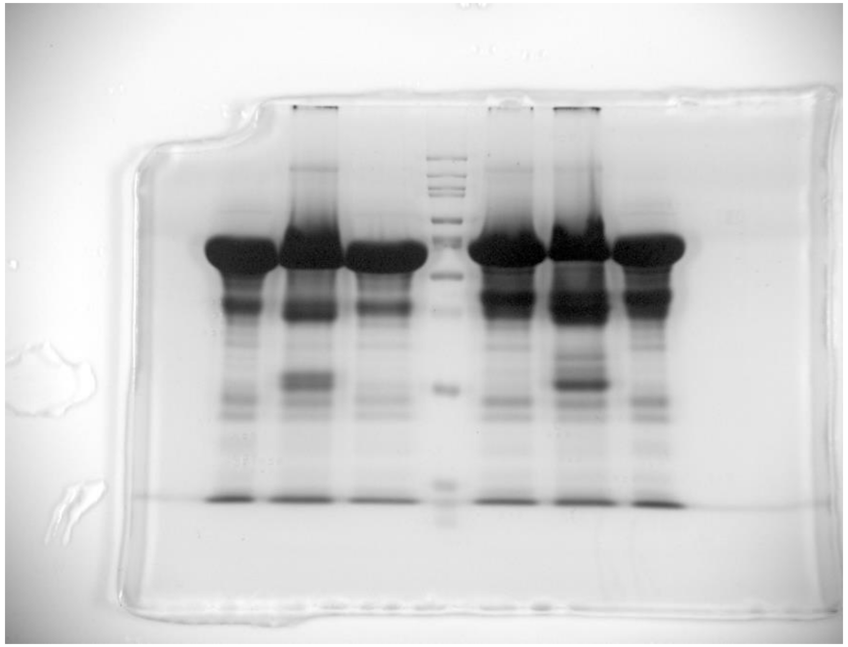

| | Substrate | Enzyme | Incubation (h) |
|---|---|---|---|
| **1.** | **PR/IN_10aa_wt** | **-** | **0** |
| **2.** | **PR/IN_10aa_wt** | **+** | **16** |
| **3.** | **PR/IN_10aa_wt** | **-** | **16** |
| **X. Molecualr weight standard** (Prosieve QuadColor) | | | |
| **5.** | **PR/IN_20aa_wt** | **-** | **0** |
| **6.** | **PR/IN_20aa_wt** | **+** | **16** |
| **7.** | **PR/IN_20aa_wt** | **-** | **16** |

**Figure 5B.**

Unedited original gel image.

To prepare Figure 5B, bands were visualized in the same gel using three different methods. Fluorescence was detected by UV or Blue light transillumination, while image for Coomassie-stained gels were taken under visible light.

Here a **Coomassie-stained** gel is shown, the image was taken by AlphaImager HP system (Protein Simple, USA).

**1 2 3 X 5 6 7 X**

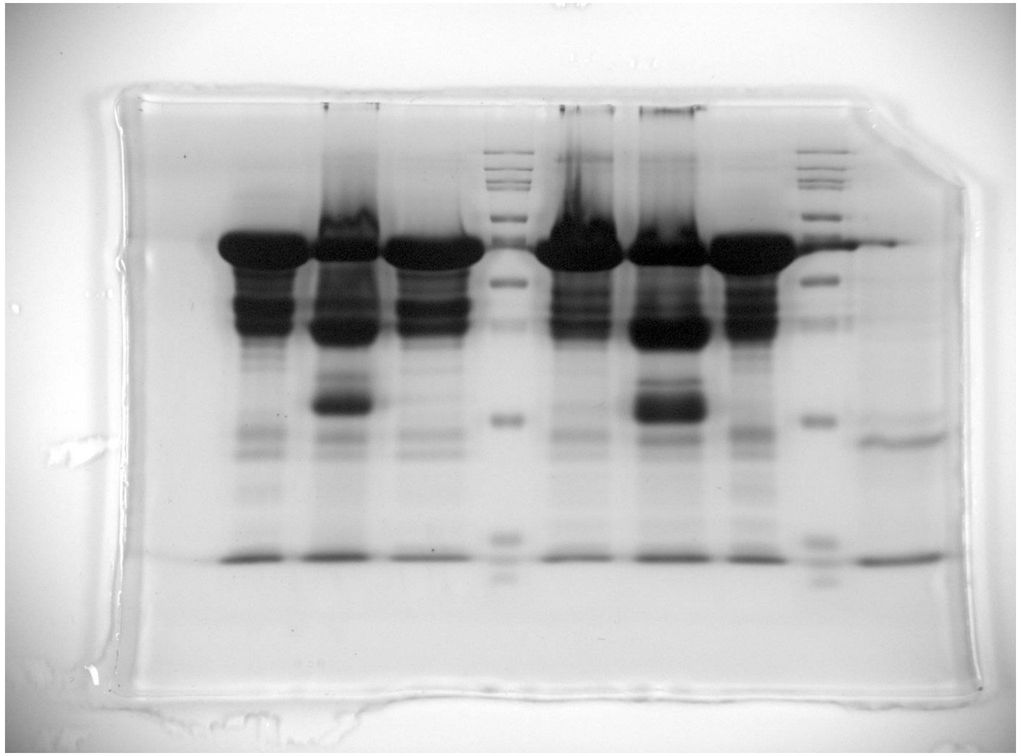

| | Substrate | Enzyme | Incubation (h) |
|---|---|---|---|
| 1. | GAG/PR_20aa_wt | - | 0 |
| 2. | GAG/PR_20aa_wt | + | 16 |
| 3. | GAG/PR_20aa_wt | - | 16 |

**X. Molecualr weight standard** (Prosieve QuadColor)

| | | | |
|---|---|---|---|
| 5. | IN/RT_20aa_wt | - | 0 |
| 6. | IN/RT_20aa_wt | + | 16 |
| 7. | IN/RT_20aa_wt | - | 16 |

**X. Molecualr weight standard** (Prosieve QuadColor)
